# Interleukin 2 modulates thymic-derived regulatory T cell epigenetic landscape

**DOI:** 10.1038/s41467-018-07806-6

**Published:** 2018-12-18

**Authors:** Laurent Chorro, Masako Suzuki, Shu Shien Chin, Tere M. Williams, Erik L. Snapp, Livia Odagiu, Nathalie Labrecque, Grégoire Lauvau

**Affiliations:** 1 Department of Microbiology and Immunology, Albert Einstein College of Medicine, 1301 Morris Park Avenue, Bronx, NY 10461 USA; 2 Department of Genetics, Albert Einstein College of Medicine, 1301 Morris Park Avenue, Bronx, NY 10461 USA; 3 Department of Anatomy and Structural Biology, Albert Einstein College of Medicine, 1301 Morris Park Avenue, Bronx, NY 10461 USA; 4 Maisonneuve-Rosemont Hospital Research Center and Department of Medicine and Microbiology, Immunology and Infectiology, University of Montreal, 5345 Boulevard de l’Assomption, Montréal, QC H1T 4B3 Canada; 5 Present Address: Janelia Research Campus of the Howard Hughes Medical Institute, Ashburn, VA 20147 USA

## Abstract

Foxp3^+^CD4^+^ regulatory T (T_reg_) cells are essential for preventing fatal autoimmunity and safeguard immune homeostasis in vivo. While expression of the transcription factor Foxp3 and IL-2 signals are both required for the development and function of T_reg_ cells, the commitment to the T_reg_ cell lineage occurs during thymic selection upon T cell receptor (TCR) triggering, and precedes the expression of Foxp3. Whether signals beside TCR contribute to establish T_reg_ cell epigenetic and functional identity is still unknown. Here, using a mouse model with reduced IL-2 signaling, we show that IL-2 regulates the positioning of the pioneer factor SATB1 in CD4^+^ thymocytes and controls genome wide chromatin accessibility of thymic-derived T_reg_ cells. We also show that T_reg_ cells receiving only low IL-2 signals can suppress endogenous but not WT autoreactive T cell responses in vitro and in vivo. Our findings have broad implications for potential therapeutic strategies to reprogram T_reg_ cells in vivo.

## Abstract (web summary)

Regulatory T (Treg) cells are developed in the thymus, and are essential for suppressing detrimental autoimmunity. Here the authors show, using mice with dampened interleukin 2 (IL-2) signaling, that IL-2 helps position the pioneer factor SATB1 to control genome-wide chromatin accessibility to facilitate T_reg_ cell lineage commitment in the thymus.

## Introduction

Naturally occurring, thymus-derived Foxp3^+^ T_reg_ cells represent a distinct lineage of CD4^+^ T cells which major role is to maintain self-tolerance^1–4^. Foxp3, a forkhead/winged helix X-linked transcription factor (TF), is the major lineage-specifying TF for these cells and is indispensable for their differentiation, long-term maintenance, and suppressive functions^5–7^. Functional loss of FOXP3 is associated with the rapid onset of fatal T cell-mediated autoimmunity, also known as the IPEX syndrome in humans (immune dysregulation, polyendocrinopathy, enteropathy, X-linked^8^) and the Scurfy phenotype in mice^5–7^. Foxp3 is induced in thymocytes undergoing positive selection following T cell receptor (TCR) triggering^9,10^ and additional signals such as IL-2, which stabilizes Foxp3 and the associated thymic-derived T_reg_ cell program of differentiation^11–13^. The transcriptional regulation of Foxp3 is complex involving the cooperation with multiple additional TFs to enable and maintain T_reg_ cell functional attributes^10,12,14–16^. Foxp3 therefore acts as an essential TF, which sustained expression is regulated by three intronic conserved non-coding sequence (CNS) elements (CNS1-3)^17^. CNS3 for instance, acts as a pioneer element in inducing the expression of Foxp3 while CNS2 is bound by IL-2-activated STAT5, directly enabling the stabilization of Foxp3 expression in T_reg_ cells^18,19^. The lack of IL-2, its high affinity receptor chain IL-2Rα/CD25 or its transducing chain IL-2Rβ/CD122^20^, lead to the development of wasting autoimmunity as a result of the loss of stable Foxp3 expression^5–7^ and subsequent Foxp3^+^ T_reg_ cells in the periphery^21–23^.

While the Foxp3 TF is required to maintain peripheral T_reg_ cell identity and functions, it is not sufficient per se to confer the functional attributes of T_reg_ cells^12,16,24^, also consistent with two-thirds of the T_reg_ cell transcriptional signature that cannot be induced by ectopic expression of Foxp3 in T_conv_ cells^14,25^. Studies using *Foxp3*^*Gfp*^ knock-in/out reporter mice in which GFP^+/+^ thymocytes lacked functional Foxp3, showed that Foxp3 acts to amplify and fix pre-established molecular features of T_reg_ cells acquired during thymic selection but prior Foxp3 expression^14^. *Foxp3*^*Gfp/Gfp*^ thymocytes exhibit CpG hypomethylation patterns characteristic of mature peripheral T_reg_ cells^24^. Foxp3 also overwhelmingly binds to pre-existing genome-wide enhancers in thymocytes committed to become T_reg_ cells during positive selection^26^. Altogether these findings suggest that TCR-induces epigenetic modifications independently of Foxp3 but likely to involve other transcriptional regulators predefining T_reg_ cell identity. Two recent reports provided mechanistic evidence for this concept^27,28^ by showing (i) that the epigenetic modifier SATB1 is essential in activating T_reg_ cell-specific super-enhancers associated with *Foxp3* and other T_reg_-cell signature genes in thymic precursor T_reg_ cells^27^ and (ii) that the methylation enzyme MLL4, which regulates the level of monomethylated H3K4 and chromatin interactions at putative gene enhancers, sets the enhancer landscape for Foxp3 induction via chromatin looping^28^.

These studies represent important conceptual advances in our understanding of the molecular genetics underlying T_reg_ cell-lineage commitment. However, other signals subsequent or concomitant to TCR triggering that may contribute to setting up the functional identity of Foxp3^+^ T_reg_ cells are largely unknown. IL-2 is proposed to be essential in this process^12,19,21,29^, but it is believed to be through the stabilization of the Foxp3 TF. Since TCR signaling leads to CD25 upregulation on thymocytes, it is conceivable that IL-2 contributes to establishing thymic T_reg_ cell identity in vivo.

Herein, we isolate and characterize a mouse model, the *Il2ra*^*mut/mut*^ mouse that bears a point mutation in the IL-2 receptor high affinity chain CD25 resulting in a selective and quantifiable decrease in response to only high affinity IL-2 signals, to test the hypothesis that IL-2 signals modulate T_reg_ cell epigenetic and transcriptional identity, and subsequent suppressive functions in vivo. Our results suggest that IL-2, possibly through the positioning of the genome organizer SATB1, modulates thymic-derived T_reg_ cell epigenetic identity prior to Foxp3 expression. T_reg_ cells that integrate low IL-2 signals (*Il2ra*^*mut/mut*^) only repress endogenous but not WT autoreactive T cells, illustrating further the importance of IL-2 signaling for optimal T_reg_ cell functions. These observations are consistent with the idea that altering T_reg_ cell epigenetic identity, in addition to IL-2 capture and signaling, leads to more rapid autoimmunity, and further raise the possibility that epigenetic reprogramming of T_reg_ cells at the time of their selection in the thymus could improve T_reg_ cell functions in autoimmune patients.

## Results

### Isolation of a novel IL-2 receptor alpha point mutant mouse

**Fig. 1:**
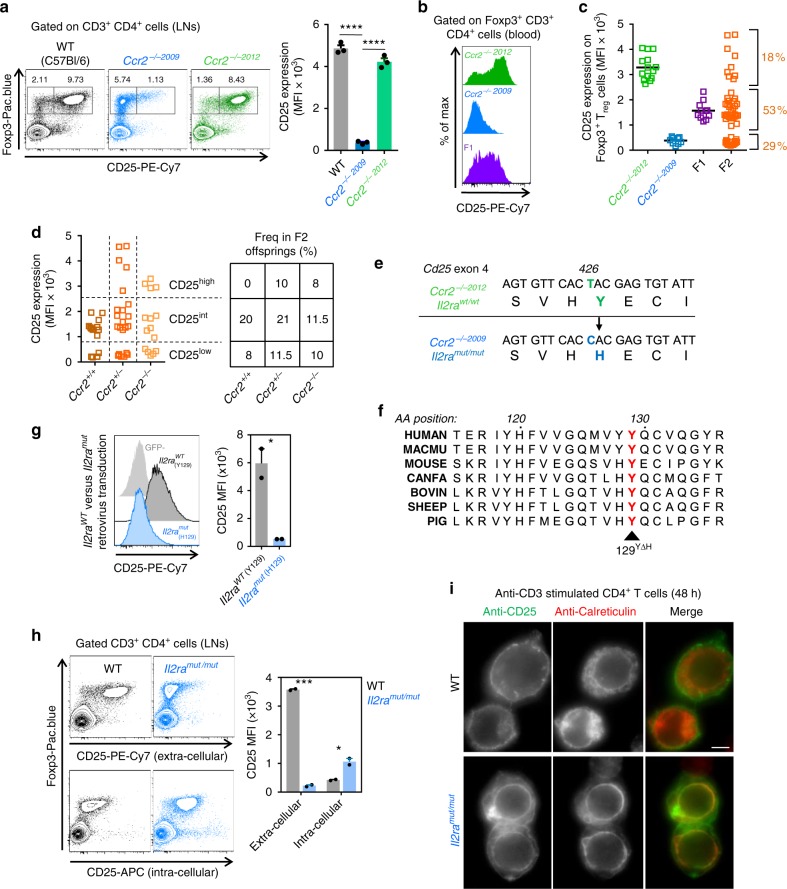
Isolation and characterization of the *Il2ra*^*mut/mut*^ mouse model. **a** Lymph node cells isolated from WT, *Ccr2*^−*/*−*2009*^ and *Ccr2*^−*/*−*2012*^ (all on the B6 background) mice were stained with mAbs against cell-surface CD3, CD4, CD25 (clone PC61), and intracellular Foxp3. A representative dot plot is shown. Bar graph summarizes CD25 expression levels (MFI) across all mice analyzed. **b** FACS histograms of cell-surface CD25 levels (MFI) gated on blood Foxp3^+^ T_reg_ cells (CD3^+^CD4^+^) from *Ccr2*^−*/*−*2009*^, *Ccr2*^−*/*−*2012*^*, Ccr2*^*+/*−*2009*^ (F1) mice. **c** Distribution of CD25 cell-surface expression levels (MFI) in T_reg_ cells from the blood of individual mice of indicated genotypes including *Ccr2*^*+/*−*2009*^ × *Ccr2*^*+/*−*2009*^ (F2) mice. **d** *Ccr2* genotypes based on CD25 surface expression levels and experimental frequencies obtained in F2 offsprings. **e** DNA and corresponding amino-acid sequence alignments of the mutation found in exon 4 of *Il2ra* gene in *Ccr2*^−*/*−*2009*^ vs *Ccr2*^−*/*−*2012*^ mice after whole-exome sequencing. **f** Alignment of the CD25 amino-acid sequence surrounding tyrosine 129 across multiple species. **g** FACS histograms of cell-surface expression of CD25 in 293T cells retrovirally transduced with WT or mutagenized Y129H *Il2ra* cDNA. Bar graph shows CD25 expression (MFI) across two independent transduction experiments. **h** Dot plots of cell-surface or intracellular expression levels (MFI) of CD25 in WT and mutant T_reg_ cells. Bar graphs average data from one of 2–3 experiments with similar results with >2 mice per group. **i** Fluorescent microscopy staining of CD25 on anti-CD3 stimulated purified CD4^+^ T cells isolated from LNs. T cells were fixed and co-stained with anti-CD25 (green) and anti-calreticulin (red) prior to image acquisition. Scale bar is 5 μm. *p*-values are indicated when relevant with *p < 0.05; **p < 0.01; ***p < 0.001; NS not significant, using two-tailed unpaired Student’s *t-*test. Error bars are mean ± SEM in all figures

During experiments in *Ccr2*^−/−^ mice^30^ obtained from the Jackson Laboratory (JacksLab) in 2009, referred to as *Ccr2*^−*/*− 2009^, we observed that CD4^+^ Foxp3^+^ T_reg_ cells in primary and secondary lymphoid organs and blood, displayed low to no cell-surface expression of the IL-2Rα/CD25 compared to T_reg_ cells from wild type (WT) B6 counterparts (WT) (Supplementary Fig. 1a). T_reg_ cell frequency among CD4^+^ T cells was reduced in spleens and lymph nodes (LNs) while remaining comparable in blood and primary lymphoid organs (Supplementary Fig 1b, c). Since *Ccr2*^−*/*−^ mice were neither reported to have different T_reg_ cell frequencies nor distinct expression of CD25 compared to WT mice^31,32^, we re-acquired *Ccr2*^−*/*−^ mice from the JacksLab in 2012, referred to as *Ccr2*^−*/*− 2012^, and compared them to *Ccr2*^−*/*− 2009^ mice (Fig. 1a). As previously documented, and in contrast to *Ccr2*^−*/*− 2009^ mice, T_reg_ cell frequencies and CD25 expression were unaltered in *Ccr2*^−*/*− 2012^ mice. We reasoned that the altered T_reg_ cell phenotype could be accounted for by (i) a difference in the skin and/or gut microbiota between the two *Ccr2*^−*/*−^ lines, or (ii) a spontaneous mutation acquired in the *Ccr2*^−*/*− 2009^ colony before 2009. Between 2009 and 2012, *Ccr2*^−*/*− 2009^ mice were backcrossed twice to the WT B6 background and underwent 23 intercrosses (JacksLabs communication). Co-housing of *Ccr2*^−*/*− 2012^ and *Ccr2*^−*/*− 2009^ mice for 3 months neither rescued T_reg_ cell frequencies nor expression of CD25 in *Ccr2*^−*/*− 2009^ mice, likely ruling out a microbiota hypothesis (Supplementary Fig. 1d). However, intercross of *Ccr2*^−*/*− 2009^ and WT B6 mice revealed that T_reg_ cells in the F1 offspring expressed twice as less cell-surface CD25 (MFI) compared to *Ccr2*^−*/*− 2012^ T_reg_ cells, but twice as more compared to *Ccr2*^−*/*− 2009^ T_reg_ cells, suggesting that the *Ccr2*^−*/*− 2009^ trait is haploinsufficient (Fig. 1b, c). Analysis of CD25 expression levels on T_reg_ cells from 51 F2 offspring obtained from intercrossing F1 mice revealed a quasi-Mendelian distribution of the various possible phenotypes, consistent with the *Ccr2*^−*/*− 2009^ trait being controlled by a single gene. Plotting CD25 expression as a function of CCR2 genotypes in the F2 progeny established that CCR2 and CD25 segregated independently of each other with the number of meiosis events close to 50% (Fig. 1d and Supplementary Fig. 1e). This result suggests that the *Ccr2*^−*/*− 2009^ trait depends on a single locus likely located on a distinct chromosome than the 9th that carries *Ccr2*. Whole exome sequencing of *Ccr2*^−*/*− 2009^ and *Ccr2*^−*/*− 2012^ mouse DNA identified only 5 non-synonymous coding gene variants (Fig. 1e and Supplementary Fig. 1f), with the most relevant coding variant replacing a single thymidine with a cytosine nucleotide on position 426 of exon 4 of the *Il2ra* gene in the *Ccr2*^−*/*− 2009^ line. This mutation led to (i) the replacement of an evolutionarily highly conserved tyrosine (Y) with a histidine (H) on position 129 of the CD25 protein (Fig. 1f), and (ii) the creation a new BssSI restriction site (Supplementary Fig. 1g) which helped confirm the presence of this mutation in *Ccr2*^−*/*− 2009^ but not *Ccr2*^−*/*− 2012^ mice. Targeted mutagenesis of the Y129 of WT *Il2ra* to H129 and retroviral transduction of WT and H129 CD25 in 293T cells (Fig. 1g) recapitulated the CD25 cell-surface expression phenotypes reported in *Ccr2*^−*/*− 2009^ vs *Ccr2*^−*/*− 2012^ mice, demonstrating that this point mutation accounted for the mouse phenotype. We next bred mice carrying only the homozygous Y129H mutation from the *Ccr2*^*+/+*^ F2 progeny (Fig. 1c), further referred to as *Il2ra*^*mut/mut*^ mice.

Since the Y129H change impaired cell-surface expression of CD25 on T_reg_ cells, we further hypothesized that CD25 may not be properly folded and/or lack stability. Based on the structure of human CD25, which has ~60% percent homology with mouse CD25, the Y129 likely makes stabilizing hydrogen bonds with a section of CD25 that is in direct contact with IL-2 (Supplementary Fig. 1h). Consistent with this possibility, intracellular staining for CD25 revealed high levels of CD25 inside T_reg_ cells from *Il2ra*^*mut/mut*^ compared to WT mice, a finding also confirmed in retrovirally transduced 293T cells (Fig. 1h and Supplementary Fig. 1i). CD25 subcellular localization in anti-CD3-stimulated CD4^+^ T cells from either *Il2ra*^*mut/mut*^ or WT mice, suggested that mutant CD25 mostly co-localized with calreticulin, an endoplasmic reticulum (ER) resident protein. A strong staining of the nuclear envelope, a subdomain of the ER, was observed in *Il2ra*^*mut/mut*^ but not WT CD4^+^ T cells, suggesting that the Y129H mutation prevented proper folding of CD25 and subsequent egress from the ER to the cell surface (Fig. 1i). Thus, we isolated and characterized a mouse mutant line with a point mutation in an evolutionarily highly conserved tyrosine of the high affinity IL-2 receptor alpha chain CD25, likely preventing its optimal folding and subsequent cell-surface expression.

### *Il2ra*^*mut/mut*^ T cells exhibit impaired responses to IL-2

**Fig. 2:**
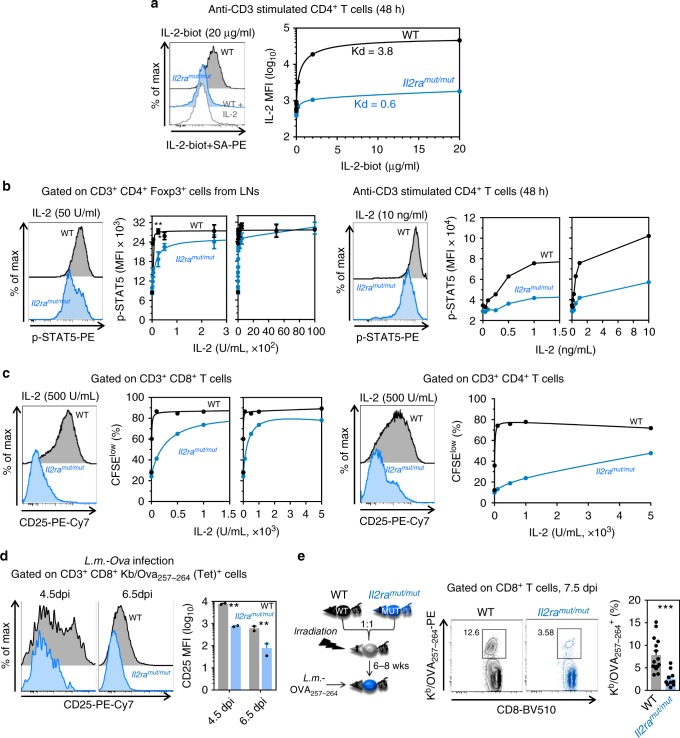
The Y129H mutation in CD25 impairs IL-2 binding, signaling and T cell proliferation. **a** Recombinant human IL-2 was biotinylated and incubated with anti-CD3 stimulated purified CD4^+^ T cells from *Il2ra*^*mut/mut*^ or WT mice prior to staining with streptavidin PE. Unbiotinylated IL-2 was added to WT T cells as a control of binding specificity. Representative FACS histograms of IL-2 or no IL-2 bound to mutant or WT T cells are shown. The graph plots IL-2 binding signals (MFI) with increasing concentration of biotinylated IL-2. IL-2 Kd (50% binding capacity) for *Il2ra*^*mut/mut*^ or WT T cells are shown. **b** Purified CD4^+^ T cells from LNs of *Il2ra*^*mut/mut*^ or WT mice (*n* = 3) were incubated in serum-free media (30’) and with IL-2 (20’) before proceeding to intracellular STAT5 phosphorylation staining. FACS histograms of STAT5 phosphorylation levels in Foxp3^+^ T_reg_ (left) and anti-CD3 activated CD4^+^ T_conv_ (right) cells are shown. Graphs plot phosphorylated STAT5 expression with increased IL-2 concentrations. **c** Purified LN CD4^+^ or CD8^+^ T cells from *Il2ra*^*mut/mut*^ or WT mice were CFSE-labeled and incubated with anti-CD3 and IL-2. The proportion of CFSE^low^ T cells was determined by FACS 4 days later. Graphs plot the % CFSE^low^ T cells as IL-2 increases in 1 of 3 replicate experiments. FACS histograms show cell-surface CD25 expression in *Il2ra*^*mut/mut*^ or WT T cells 4 days post IL-2 incubation. **d** *Il2ra*^*mut/mut*^ or WT mice (*n* = 2) were immunized i.v. with 10^4^ WT *Listeria monocytogenes (Lm)* expressing Ovalbumin (*Lm*-Ova) and spleen cells were stained at indicated days with Ova_257-264_/K^d^ tetramers (Tet^+^) and anti-CD25. Representative FACS histograms of CD25 expression on tet^+^ CD8^+^ T cells is shown with a summary bar graph. **e** WT/*Il2ra*^*mut/mut*^ mixed bone-marrow (BM) chimeras (ratio 1:1) with discriminative CD45.1/2 congenic markers were immunized i.v. with WT *Lm-Ova* and 7.5 days later spleen cells stained with Ova_257-264_/K^d^ tetramers. Representative FACS dot plots and summary bar graphs of *Lm*-specific *Il2ra*^*mut/mut*^ or WT tet^+^ CD8^+^ T cells are shown (*n* = 15 mice). *p*-values are indicated with *p < 0.05; **p < 0.01; ***p < 0.001; NS not significant, using two-tailed unpaired Student’s *t*-test. Error bars are mean ± SEM in all figures

We next assessed whether impaired cell-surface expression of CD25 on T cells translates into decreased IL-2 binding and signaling. Using labeled IL-2 incubated with anti-CD3 stimulated CD4^+^ T cells, we found higher IL-2 binding to WT compared to either *Il2ra*^*mut/mut*^ or WT T cells incubated with an excess of non-biotinylated IL-2 (Fig. 2a and Supplementary Fig. 2a). STAT5a phosphorylation, a very early signaling response that follows IL-2 binding to CD25, was also profoundly altered both in T_reg_ cells from thymus and LNs and in conventional CD4^+^ T (T_conv_) cells from *Il2ra*^*mut/mut*^ mice (Fig. 2b and Supplementary Fig. 2a, b). Consistent with these findings, *Il2ra*^*mut/mut*^ CD8^+^ and CD4^+^ T_conv_ cells proliferated substantially less compared to WT counterparts as quantified in vitro by CTV dilution of labeled T_conv_ cells after anti-CD3 and varied amounts of IL-2 (Fig. 2c and Supplementary Fig. 2a, c). To extend findings in vivo, we next monitored CD25 upregulation on K^b^/Ova_257-264_ tetramer^+^ (Tet^+^) CD8^+^ T_conv_ cells primed in WT or *Il2ra*^*mut/mut*^ mice infected intravenously (i.v.) with *Listeria monocytogenes* expressing the model antigen Ovalbumin (*Lm-Ova*) (Fig. 2d). Expression of CD25 by Ova-specific Tet^+^ CD8^+^ T_conv_ cells was lower in *Il2ra*^*mut/mut*^ compared to WT mice^33^, in line with the in vitro data (Fig. 2d). Then, we generated mixed bone-marrow chimeras from lethally irradiated recipient mice reconstituted with WT and *Il2ra*^*mut/mut*^ donor bone-marrow cells (ratio 1:1) so that we could monitor and compare WT and *Il2ra*^*mut/mut*^ antigen-specific CD8^+^ T_conv_ cell responses in the same host (Fig. 2e and Supplementary Fig. 2d). Reconstituted chimeras were further infected with *Lm-Ova* or the Herpes Simplex Virus 2 (HSV-2) intravaginally (i.vag.), and both K^b^/Ova_257-264_ and K^b^/gB_498-505_ Tet^+^ T_conv_ cells were monitored at the peak of the primary response. In both models, Ova- and gB-specific *Il2ra*^*mut/mut*^ CD8^+^ T_conv_ cells expanded ~3-fold less than WT counterparts. Thus, the CD25 Y129H mutation profoundly alters CD25 expression and functional response to IL-2 in both T_conv_ and T_reg_ cells.

### IL-2 modulates T_reg_ cell epigenetic landscapes

**Fig. 3:**
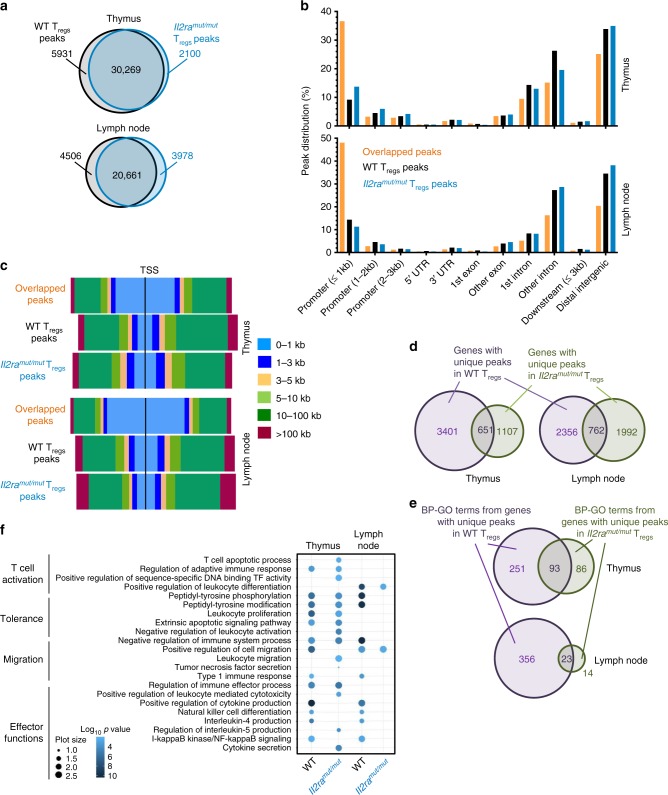
IL-2 makes substantial modifications in the epigenetic landscape of T_reg_ cells: T_reg_ cells (5 × 10^4^) were sorted by flow cytometry from thymus or LNs of *Il2ra*^*mut/mut*^ and WT *Foxp3*^*Rfp/Rfp*^ reporter mice, lysed and DNA from nucleus extracted for analysis of open chromatic regions by ATAC-seq. **a** Venn diagram of number of common and unique ATAC-seq peaks (OCRs) in *Il2ra*^*mut/mut*^ vs WT T_reg_ cells from thymus and LNs. **b**, **c** Distribution of common and unique ATAC-seq peaks across gene organization (**b**) and distance to the transcription start site (TSS, **c**) in the whole genome. **d**, **e** Venn diagrams of number of genes (**d**) and GO pathways (**e**) exhibiting unique peaks in *Il2ra*^*mut/mut*^ vs WT thymic and LN T_reg_ cells on all (**d**) or within (**e**) 20 kb from TSS. **f** Network analysis of biological-process GO term enrichment among genes with unique peaks within 20 kb from TSS in *Il2ra*^*mut/mut*^ or WT thymic and LN T_reg_ cells. Genes were analyzed for over-represented GO terms using ReviGO. Node color is proportional to the FDR-adjusted *p*-value of the enrichment. Node size is proportional to gene set size

IL-2 signals are postulated to contribute to establishing the T_reg_ cell program of differentiation before T_reg_ cells express Foxp3 in the thymus^11–13,34^ but only little evidence exist to support this hypothesis. To explore this possibility, we took advantage of the *Il2ra*^*mut/mut*^ mouse model in which T_reg_ cells receive reduced IL-2 signals, and asked whether their chromatin accessibility differs just after they have committed to the T_reg_ cell lineage (thymus) and in the periphery in the secondary lymphoid organs (LNs). We isolated Foxp3^+^ T_reg_ cells from thymus and LNs of *Il2ra*^*mut/mut*^ and WT *Foxp3*^*Rfp/Rfp*^ reporter mice and conducted an analysis of the genome-wide open chromatin regions (OCRs) using an assay for transposase accessible chromatin with high-throughput sequencing (ATAC-seq^35^, Fig. 3 and Supplementary Fig. 3). While we found 36,200 and 32,369 OCRs (ATAC-seq peaks) in WT and *Il2ra*^*mut/mut*^ thymic T_reg_ cells respectively, LN T_reg_ cells exhibited 25,167 and 24,639 OCRs (Fig. 3a). Ninety-five percent of WT LN T_reg_ cell OCRs identified overlapped with those reported in a prior study analyzing LN T_reg_ cell OCRs^36^ (Supplementary Fig. 3a). As expected, this overlap was lower (75-80%) when we compared WT T_reg_ cell’s OCRs to that reported in CD8^+^ T cells^37^. Within the thymic and LN T_reg_ cell OCRs, we respectively identified 8031 and 8484 differentially accessible OCRs in WT (5931 in thymus and 4506 in LN) and *Il2ra*^*mut/mut*^ (2100 in thymus and 3978 in LN) T_reg_ cells (Fig. 3a). This represents ~41% difference in the epigenetic landscape of WT vs *Il2ra*^*mut/mut*^ LN T_reg_ cells, which is comparable to what is reported between resting and activated T_reg_ cell OCRs^36^ (~36%, Supplementary Fig. 3b). These differences are also similar to those measured between effector and memory or exhausted CD8^+^ T cells (34–41%)^37–39^ but substantially greater than that reported in effector cells isolated from mice infected with acute vs chronic LCMV (~15%)^38^. Analysis of the relative localizations of differential OCRs in relation to the mouse ensemble annotated genes revealed a lower proportion of OCRs in the transcription start sites (TSS, ≤1 kb) compared to that of the overlapping peaks both for thymic and LN T_reg_ cells (~9–14% vs 37–48%, Fig. 3b, c and Supplementary Fig. 3c and Data 1). The majority of unique peaks were found in introns and distal intergenic regions (>10 kb), suggesting that IL-2 signals regulate chromatin opening of elements distal to the TSS already at early stages of T_reg_ cell commitment in the thymus. Upon assigning these peaks to the closest gene TSS, 651 (thymus) and 762 (LN) genes with differential OCRs were common to WT and *Il2ra*^*mut/mut*^ T_reg_ cells while the remaining were within non-overlapping genes (Fig. 3d and Supplementary Data 2). Approximately three times as many genes and OCRs were uniquely present in WT compared to *Il2ra*^*mut/mut*^ thymic T_reg_ cells (3401 vs 1107 genes) while LN T_reg_ cells had comparable numbers of unique genes and OCRs (2356 vs 1992 genes)(Fig. 3a,d). This result shows that the ratio of differential OCRs between WT and *Il2ra*^*mut/mut*^ T_reg_ cells from the thymus to the LNs is substantially modulated (from ~3:1 to 1:1), suggesting further functional commitment of T_reg_ cells. Analysis of the biological-process (BP) gene-ontology (GO) pathways of genes containing unique peaks (<20 kb from TSS) in WT thymic and LN T_reg_ cells highlighted a much greater diversity of BP (251 and 356 GO pathways vs only 86 and 14 GO pathways, respectively)(Fig. 3e and Supplementary Data 3). Since the differences in the number of genes with unique peaks between WT and *Il2ra*^*mut/mut*^ T_reg_ cells is much smaller compared to that of the GO pathways (Fig. 3d, e), it further suggests that the OCRs in *Il2ra*^*mut/mut*^ T_reg_ cells are more random. While some processes related to T cell activation and tolerance are common to both types of T_reg_ cells in the thymus, others targeting activation, migration and effector mechanisms are only maintained in WT LN T_reg_ cells (Fig. 3f). Conducting the GO pathway analysis on genes containing unique peaks without any limit from TSS revealed the exact same trend (Supplementary Fig. S3d,e and Data 3). Thus, IL-2 signals have a broad impact on genome-wide T_reg_ cell epigenetic landscape, both for the opening and the closing of mostly TSS-distant regulatory regions. IL-2-mediated alterations occur already at early stages of T_reg_ cell commitment to this lineage in the thymus and are sustained throughout LN T_reg_ cells.

### IL-2-driven epigenetic changes mildly alter gene expression

**Fig. 4:**
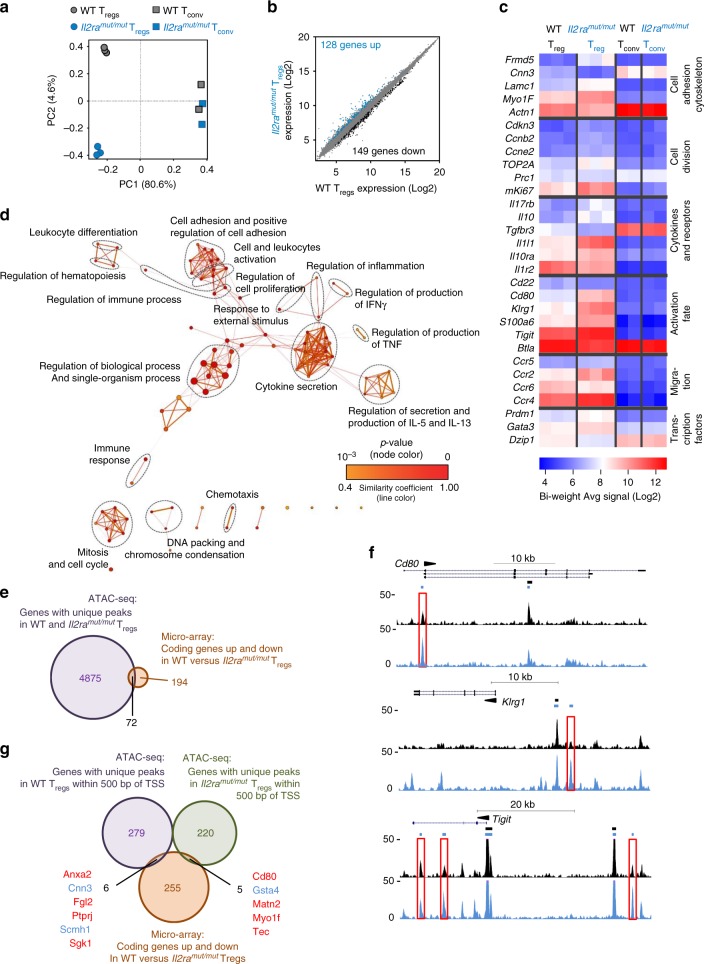
The expression of only a small set of genes in T_reg_ cells is altered by low IL-2 signals: **a** 5 × 10^4^ T_reg_ and T_conv_ cells were sorted by flow cytometry from LNs of three independent replicate *Il2ra*^*mut/mut*^ or WT *Foxp3*^*Rfp/Rfp*^ reporter mice and total RNA extracted and reverse transcribed to cDNA. Affymetrix mouse expression arrays (Pico 1.0) were then conducted. Principal component analysis (PCA) of expressed genes using the top 10% of genes with the highest variance in analyzed groups with each symbol featuring one mouse. **b** Scatter plot of individual genes expressed in *Il2ra*^*mut/mut*^ vs WT T_reg_ cells. Significantly upregulated and downregulated genes, defined as genes with at least 1.5 fold change, *p*-value ≤ 0.01, are colored blue or black, respectively, and numbers are shown. **c** Heat map of selected genes grouped under various categories as indicated and for which expression is significantly different between *Il2ra*^*mut/mut*^ and WT T_reg_ cells. **d** Network analysis of biological-process gene-ontology (GO) term enrichment among significantly upregulated genes in *Il2ra*^*mut/mut*^ vs control WT T_reg_ cells. Upregulated genes were analyzed for over-represented GO terms using BiNGO in Cytoscape, and the resulting network was calculated and visualized using EnrichmentMap. Groups of similar GO terms were manually circled. Line thickness and color are proportional to the similarity coefficient between connected nodes. Node color is proportional to the FDR-adjusted p-value of the enrichment. Node size is proportional to gene set size. **e** Venn diagram comparing differentially expressed genes in *Il2ra*^*mut/mut*^ vs WT T_reg_ cells to genes with unique peaks called in *Il2ra*^*mut/mut*^ and WT T_reg_ cells found in ATAC-seq in Fig. 3 based on gene symbols. **f** ATAC-seq tracks for 3 sample genes with at least 1 unique peak localized in distinct regions of the gene (promotor, intronic, intergenic). **g** Three-way Venn diagram comparing differentially expressed genes in *Il2ra*^*mut/mut*^ versus WT T_reg_ cells to genes containing unique peaks close to TSS (<500 bp) in *Il2ra*^*mut/mut*^ vs WT T_reg_ cells found in ATAC-seq

Since IL-2 induces extensive modifications in the chromatin of T_reg_ cells (Fig. 3), we next assessed whether these alterations translated into a distinct program of expression between *Il2ra*^*mut/mut*^ and WT LN T_reg_ cells. The lack of IL-2 signaling in T_reg_ cells was reported to only alter a relatively focused set of genes implicated in cell growth, metabolism, motility and adhesion, preventing their overall competitive fitness in vivo^21,29,40^. We purified total RNA from flow-sorted Foxp3^+^ (RFP^+^) T_reg_ or naive (CD62L^hi^CD44^lo^) T_conv_ cells isolated from the LNs of WT or *Il2ra*^*mut/mut*^*Foxp3*^*Rfp/Rfp*^ reporter mice, and analyzed gene expression using microarrays (Fig. 4). While the expression profiles of naive T_conv_ cells are almost identical between the two groups, that of *Il2ra*^*mut/mut*^ and WT T_reg_ cells exhibit significantly more differences (Fig. 4a and Supplementary Fig. 4a and Data 4). We note a set of 277 differentially expressed genes among which 128 and 149 are up- or downregulated respectively in *Il2ra*^*mut/mut*^ compared to WT T_reg_ cells (Fig. 4b and Supplementary Fig. 4b). These genes are involved in cellular adhesion and cytoskeleton rearrangement, cell division and differentiation, cytokines and receptors, cellular activation and chemotaxis (Fig. 4c), consistent with prior reports^21,29,40^. We checked some of the differentially upregulated genes for which Abs specific for encoded proteins are available, and found a direct correlation with protein expression levels for KLRG1, TIGIT, CCR4 while we did not for CCR5 or BTLA (Supplementary Fig. 4c). Interestingly, increased expression of these markers is detected only in a small subset of CD4^+^ T_reg_ cells, suggesting that lowering IL-2 signals promotes the onset of a subpopulation of T_reg_ cells with a slightly distinct expression program, rather than altering the whole T_reg_ cell population. Since only a few of the downregulated genes encode for proteins of known functions, we next focused primarily on sets of upregulated genes to run a comparison against known BP-GO databases (Fig. 4d and Supplementary Fig. 4d,e and Data 5). We found statistically over-represented gene sets implicated in cell adhesion, activation, proliferation/cell cycle, cellular differentiation as well as inflammatory processes, transcriptional regulators and chromatin organization/epigenetic gene regulation. While only a limited set of genes are differentially expressed in Foxp3^+^ T_reg_ cells that receive lower IL-2 signals, the biological functions that are altered seem to directly target their ability to migrate, undergo homeostatic proliferation and express suppressive effector functions. We also noted differences in expression of genes implicated in various steps of chromatin modifications (Supplementary Fig. 4f).

Most interestingly, although the number (4875) and relative proportion (~40%) of differential OCRs between *Il2ra*^*mut/mut*^ and WT LN T_reg_ cells is important (Fig. 3), those of differentially expressed genes (266) remain very small (below 1% of genes), suggesting that IL-2 signals play a more prominent role in shaping the epigenetic landscape and identity of T_reg_ cells than their ability to express sets of genes (Fig. 4e and Supplementary Data 6). Among the differentially expressed genes, only 72 exhibited unique OCRs in either WT or *Il2ra*^*mut/mut*^ LN T_reg_ cells. These OCRs may contribute to the changes in gene expression via chromatin structure modifications while the remaining genes (194) are likely to be controlled at the level of gene transcription with no changes in chromatin structures. For instance, CD80 that is highly expressed in *Il2ra*^*mut/mut*^ compared to WT LN T_reg_ cells, exhibits greater OCRs heights in *Il2ra*^*mut/mut*^ vs WT T_reg_ cell promoter regions (Fig. 4f). Differential OCRs are further noted in intergenic regions of KLRG1 or in intronic regions for TIGIT. Interestingly, neither the *Foxp3* nor the *Helios* genes had differential OCRs between *Il2ra*^*mut/mut*^ and WT T_reg_ cells (Supplementary Fig. 4g and Data 7). Analysis of the potential binding motifs for TFs in the differential OCRs revealed that a different set of TFs such as FOXO and KLF family members, could possibly bind in WT but not *Il2ra*^*mut/mut*^ T_reg_ cells (Supplementary Fig. 4h). Because chromatin structures in the vicinity of TSS regions play critical roles in the expression status of the gene, we tested if differentially expressed genes contain differential OCRs close to the TSS (Fig. 4g and Supplementary Data 6). Eleven differentially expressed genes contain differential OCRs close to their TSS (±500 bp) while the majority of the remaining genes did not, suggesting that these genes undergo epigenetic modifications mostly occurring on elements distal to the TSS such as gene enhancer regions. In summary, despite establishing major genome wide changes of the T_reg_ cell epigenetic landscape, IL-2 signals on LN T_reg_ cells at steady state only translate in modest modifications of their gene expression profiles.

### IL-2 regulates SATB1 positioning prior T_reg_ cell commitment

**Fig. 5:**
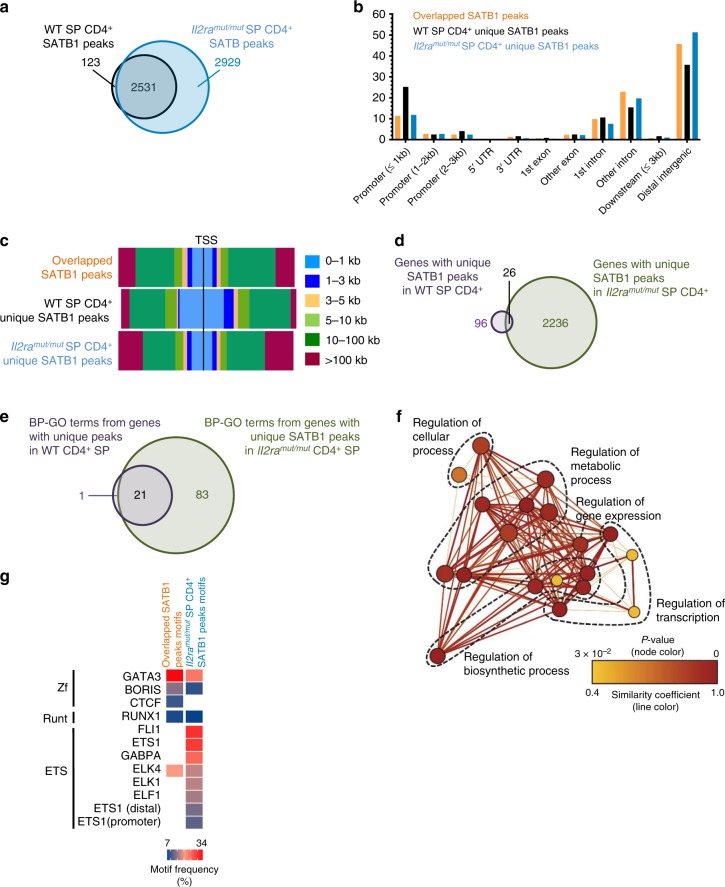
Ectopic DNA binding of SATB1 in SP CD4^+^ thymocytes receiving low IL-2 signals: 2 × 10^6^ SP CD4^+^ thymocytes were sorted in 2 independent duplicate experiments by flow cytometry from the thymus of *Il2ra*^*mut/mut*^ and WT mice, PFA fixed, lysed, and cross-linked DNA was used for chromatin immunoprecipitation sequencing (ChIP-seq) of the pioneer factor SATB1. **a** Venn diagram of the number of common and unique SATB1 DNA binding peaks in *Il2ra*^*mut/mut*^ vs WT SP CD4^+^ thymocytes. **b**, **c** Distribution of common and unique SATB1 DNA binding peaks across gene organization (**b**) and distance to the transcription start site (TSS, **c**) in the whole genome. **d** Venn diagram of the number of genes exhibiting unique SATB1 DNA binding peaks in *Il2ra*^*mut/mut*^ vs WT SP CD4^+^ thymocytes. **e** Venn diagram of biological-process gene-ontology (BP-GO) from genes with unique SATB1 DNA binding peaks in WT vs *Il2ra*^*mut/mut*^ SP CD4^+^ thymocytes. **f** Network analysis of BP-GO term enrichment among genes with unique SATB1 DNA binding peaks in WT SP CD4^+^ thymocytes. Genes with unique SATB1-binding peaks were analyzed for over-represented GO terms using BiNGO in Cytoskape, and the resulting network was calculated and visualized using EnrichmentMap in Cytoscape. Groups of similar GO terms were manually circled. Line thickness and color are proportional to the similarity coefficient between connected nodes. Node color is proportional to the FDR-adjusted *p*-value of the enrichment. Node size is proportional to gene set size. **g** Frequency of known TF binding site motifs enriched in common and unique SATB1 DNA binding peaks in WT and *Il2ra*^*mut/mut*^ SP CD4^+^ thymocytes. Motif frequency (%) is shown in heat map

We next investigated how IL-2 signals mediate such large genome-wide changes in the T_reg_ cell epigenome. Given its essential role in T_reg_ cell-lineage specification and functions^5–7,14^, and its dependence on IL-2^19,21^, one obvious candidate is the Foxp3 TF. If Foxp3 accounted for our observations, we predicted that Foxp3 binding regions would exhibit a very large overlap with the OCRs found in WT T_reg_ cells but less with that of *Il2ra*^*mut/mut*^ T_reg_ cells (Supplementary Fig. 5a). By aligning published chromatin immunoprecipitation sequencing (ChIP-seq) results of the Foxp3 TF defining the Foxp3 binding sites throughout the genome of LN T_reg_ cells^26^ with our ATAC-seq data, we found that Foxp3 DNA binding regions largely overlapped with OCRs common between *Il2ra*^*mut/mut*^ and WT LN T_reg_ cells, suggesting that IL-2-mediated alterations of the T_reg_ cell epigenome are independent of Foxp3. Robust experimental evidence supports the idea that T_reg_ cell epigenetic and functional identity is established in the thymus prior T_reg_ cells express Foxp3^13,24–26^. Notably, the pioneer factor SATB1 was reported to be essential for T_reg_ cell-lineage specification before expression of Foxp3 in the thymus but not after^27^. Thus, we hypothesized that IL-2 acts through SATB1 before thymocytes commit to the T_reg_ cell fate, at the single positive (SP) CD4^+^ thymocyte stage^11,13,24^. We know (Fig. 3) that mature Foxp3^+^ *Il2ra*^*mut/mut*^ thymic T_reg_ cells already exhibit differential OCRs compared to WT counterparts, suggesting epigenetic changes may have occurred before this stage. We conducted ChIP-seq experiments of SATB1 on SP CD4^+^ thymocytes isolated from *Il2ra*^*mut/mut*^ or WT mice, postulating that SATB1 DNA positioning would differ if indeed this pioneer factor is involved in the regulation of IL-2-mediated T_reg_ cell epigenetic changes (Fig. 5). The vast majority of SATB1-bound regions in WT and *Il2ra*^*mut/mut*^ SP CD4^+^ thymocytes overlapped. While only 123 unique DNA regions are bound by SATB1 in WT thymocytes, SATB1 associate to more than 50% additional regions (2929) in *Il2ra*^*mut/mut*^ thymocytes, showing that ectopic binding of this pioneer factor occurs when IL-2 signals are limiting (Fig. 5a). Interestingly, the 123 unique SATB1-binding regions in WT thymocytes localize closer to promoter areas compared to that of *Il2ra*^*mut/mut*^ counterparts which are far from TSS (>100 kb) in distal intergenic regions (Fig. 5b, c and Supplementary Fig. 5b). Assigning the 123 SATB1-bound regions to the nearest gene TSS highlighted 96 genes among which 26 are common to *Il2ra*^*mut/mut*^ T_reg_ cells despite different SATB1-bound areas (Fig. 5d and Supplementary Data 7). Interestingly, 29 of the 96 genes with unique SATB1-binding sites are located within less than 2 kb from the TSS, and a majority encodes for DNA remodeling proteins, cell cycle, signal transduction and metabolism (Supplementary Fig. 5c). These genes exhibit variable levels of expression across thymocyte development^41^, in particular between the SP CD4^+^ thymocyte and thymic T_reg_ cell stages, consistent with a possible regulatory role of their expression by promoter-bound SATB1. SATB1 binds ~20 times more genes (2236) in *Il2ra*^*mut/mut*^ compared to WT thymocytes, illustrating its ectopic binding when IL-2 signals are limiting (Fig. 5d). BP-GO analysis on the common genes between the two conditions revealed that SATB1 targets common processes (~21) involved in DNA remodeling, transcriptional regulation and metabolism (Fig. 5e and Supplementary Fig. 5d and Data 8a,b). However, when IL-2 signaling is impaired, SATB1-associated genes are implicated in 83 additional processes that include immune cell activation, adhesion and differentiation, and may reflect less focused binding. A search for known TF binding motifs within the regions bound by SATB1 highlighted GATA3, RUNX1 and ETS1 among the top ones, which are all associated with the regulation of Foxp3 expression^15,17,42,43^ (Fig. 5g). These motifs are AT-rich, which represent SATB1 preferential binding motifs^44^. While no enriched motifs are revealed in the 123 unique regions of WT SP CD4^+^ thymocytes, all of the prior ones are found in the remaining WT and *Il2ra*^*mut/mut*^ SATB1-bound regions. The proportion of these TF-binding motifs is enriched in peaks common with WT thymocytes, suggesting a more focused binding of SATB1 when normal IL-2 signals are received. Thus, IL-2 signals have a significant impact on where the pioneer factor SATB1 binds to DNA in SP CD4^+^ thymocytes, which is likely to contribute to IL-2 modulation of the Foxp3^+^ T_reg_ cell epigenetic landscape.

### *Il2ra*^*mut/mut*^ mice lack autoimmunity despite altered chromatin

**Fig. 6:**
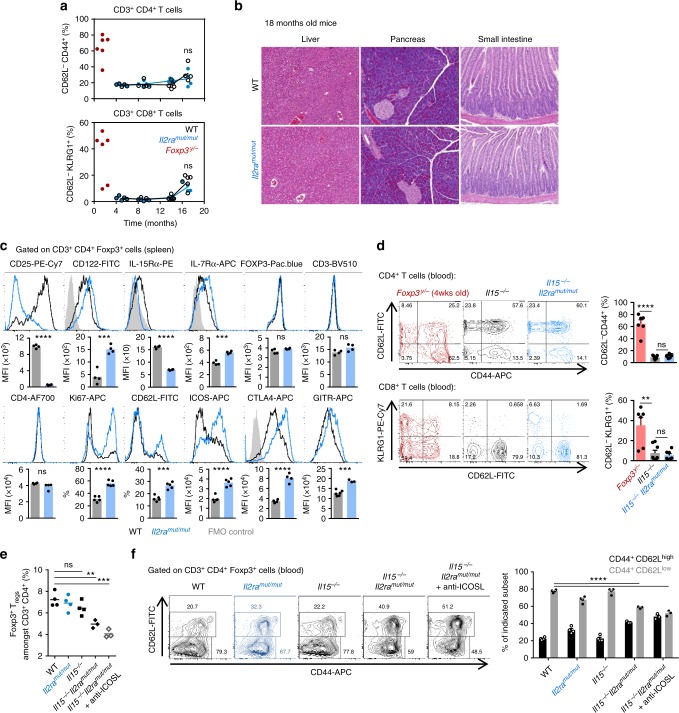
*Il2ra*^*mut/mut*^ mice do not develop any signs of autoimmunity: **a** T cells collected from blood of *Il2ra*^*mut/mut*^ and WT mice were stained for cell-surface CD3, CD8, CD4 and CD62L, CD44, KLRG1 activation markers at indicated times over an 18 months period. Blood from control *Foxp3*^*y/*−^ was analyzed at ~1.5 months post birth. Each symbol represents an individual mouse. **b** At month 18, liver, pancreas, and small intestine were harvested, fixed/cut, and stained with H/E. **c** Spleen cells from 8 to 10-weeks-old *Il2ra*^*mut/mut*^ and WT mice were stained for cell-surface CD3, CD4, intracellular Foxp3 and indicated markers. Overlay of representative FACS histograms after gating on CD3^+^CD4^+^Foxp3^+^ T cells are shown and bar graphs average relative expression levels (MFI, *n* = 4–5). **d** Blood from *Il2ra*^*mut/mut*^*Il15*^−*/*−^, *Il15*^−*/*−^ (6 months old) or *Foxp3*^*y/*−^ mice was stained as in **a** and representative FACS dot plots with bar graphs summarizing average across mice (*n* = 6–7) are shown. **e** Proportion of peripheral blood Foxp3^+^ T_reg_ cells among CD3^+^CD4^+^ T cells in indicated mice treated (or not) with anti-ICOSL for 2 weeks. **f** Blood leukocytes from indicated mice groups were stained for expression of CD3, CD4, intracellular Foxp3, CD62L, CD44. Representative dot plots and summary bar graphs of average CD44/CD62L expression levels across all groups of mice are shown (*n* = 3–4). *p*-values are indicated when relevant with *p < 0.05; **p < 0.01; ***p < 0.001; NS not significant, using two-tailed unpaired Student’s *t-*test

Given the importance of IL-2 in T_reg_ cell stability, immune homeostasis^21–23,40^ and epigenetic programming (Figs. 3, 5), we postulated that *Il2ra*^*mut/mut*^ mice may develop symptoms consistent with autoimmunity over time. Monitoring of T cell phenotypes in the peripheral blood of *Il2ra*^*mut/mut*^ and WT mice over ~1.5 years did not reveal any differences in cell-surface expression of classical activation markers (CD44, CD62L, KLRG1), in contrast to T cells from *Foxp3*^*y/*−^ mice analyzed 1.5 months post birth (Fig. 6a and Supplementary Fig. 6a). No T cell infiltrates were detected in the liver, pancreas or small intestine of *Il2ra*^*mut/mut*^ mice, which all represent peripheral organs often associated with the onset of an autoimmune reaction (Fig. 6b). We next investigated how mutant T_reg_ cells may control autoreactive T_conv_ cells and maintain immune homeostasis. We first conducted an extensive characterization of T_reg_ cells from *Il2ra*^*mut/mut*^ compared to WT mice (Fig. 6c and Supplementary Fig. 6b). *Il2ra*^*mut/mut*^ T_reg_ cells express higher cell-surface levels of IL-7 and IL-15 survival receptors (CD127 and CD122) and lower levels of IL-15Rα, consistent with the idea that T_reg_ cells receiving lower IL-2 signals may rely on the use of compensatory IL-7 and IL-15 cytokines^11,19,29^. These T_reg_ cells also undergo increased cell proliferation (Ki67^+^) and upregulated T cell inhibitory receptors (CTLA4, GITR) and ICOS, which are reported to promote efficient T_reg_ cell-mediated suppression^45^. We did not detect any difference in TCR Vβ usage (Supplementary Fig. 6c), likely ruling out major biases in *Il2ra*^*mut/mut*^ T_reg_ cell repertoire. Since the common IL-2/IL-15 transducing beta chain CD122 was significantly upregulated on *Il2ra*^*mut/mut*^ T_reg_ cells compared to WT counterparts (spleen and thymus), we next crossed *Il2ra*^*mut/mut*^ mice to *Il15*^−*/*−^ mice, postulating that IL-15 signals are essential for mutant T_reg_ cells to maintain immune homeostasis in these mice (Fig. 6d) and compensate for impaired IL-2 signals though *Il2ra*^*mut/mut*^ mice have higher levels of circulating IL-2 (Supplementary Fig. 6d). The proportion of activated CD4^+^ (CD62L^lo^CD44^hi^) and CD8^+^ (CD62^lo^KLRG1^+^) T_conv_ cells in *Il2ra*^*mut/mut*^*Il15*^−*/*−^ and *Il15*^−*/*−^ mice was equivalent (Fig. 6d) and similar to that of WT mice (Fig. 6a), but significantly lower than in *Foxp3*^*y/*−^ mice. In addition, we did not find any evidence of T cell infiltrates in the liver or the spleen of these mice (Supplementary Fig. 6e). However, the proportion of Foxp3^+^ T_reg_ cells in *Il2ra*^*mut/mut*^*Il15*^−*/*−^ mice was reduced compared to WT, *Il2ra*^*mut/mut*^ or *Il15*^−*/*−^ mice (Fig. 6e). Recently, two distinct subsets of T_reg_ cells discriminated on CD44, CD62L and CCR7 expression were proposed to require either IL-2 (CD44^lo^CD62^hi^) or ICOS (CD44^hi^CD62^lo^) signaling for their maintenance^46^. Since we noted high cell-surface expression of ICOS on *Il2ra*^*mut/mut*^ T_reg_ cells (Fig. 6c), we reasoned that impaired IL-2 signals might favor the onset of a CD44^hi^CD62^lo^ T_reg_ cell subset that requires ICOS signaling. Unexpectedly, we only observed lower proportions of the CD44^hi^CD62^lo^ T_reg_ cell subset in *Il2ra*^*mut/mut*^ and *Il2ra*^*mut/mut*^*Il15*^−*/*−^ compared to WT mice, and this proportion was even further decreased by blocking ICOS for 2 weeks (Fig. 6f). The overall proportion of Foxp3^+^ T_reg_ cells among CD4^+^ T cells in the blood of ICOS-neutralized *Il2ra*^*mut/mut*^*Il15*^−*/*−^ mice was also reduced by ~50%, although mice did not develop autoimmunity (Fig. 6e and Supplementary Fig. 6f), suggesting that none of the proposed mechanisms accounts for the absence of autoimmune disease in *Il2ra*^*mut/mut*^ mice.

### *Il2ra*^*mut/mut*^ T_reg_ cells cannot repress WT autoimmune T cells

Lack of IL-2 or its receptor chains leads to fatal autoimmunity because T_reg_ cells are unstable and fail to suppress autoreactive T_conv_ cells^11–13^. Yet, mice in which IL-2 signaling is genetically altered by targeted mutation in the IL-2Rβ/CD122 transducing chain do not become autoimmune^29^. Thus, we reasoned that similar to IL-2Rβ mutant T_reg_ cells, *Il2ra*^*mut/mut*^ T_reg_ cells may repress *Il2ra*^*mut/mut*^ but not WT autoreactive T_conv_ cells. As a first step to assess this idea, we conducted a standard in vitro suppression assay using increasing numbers of *Il2ra*^*mut/mut*^ or control WT T_reg_ cells incubated with CFSE-labeled anti-CD3 activated T_conv_ cells (Supplementary Fig. 7a). *Il2ra*^*mut/mut*^ T_reg_ cells inhibit T_conv_ cell proliferation ~two fold less than WT T_reg_ cells across all ratios, possibly accounting for the lack of autoimmunity in *Il2ra*^*mut/mut*^ mice. Since T_reg_ cells grown in vitro with anti-CD3/CD28 stimulation and under limited IL-2 availability lose Foxp3 expression and convert to effector T cells^18^, we next assessed whether *Il2ra*^*mut/mut*^ T_reg_ cells also lacked stability after TCR triggering in vitro (Supplementary Fig. 7b). While ~60% of WT T_reg_ cells lost Foxp3 expression, ~85% of *Il2ra*^*mut/mut*^ converted to Foxp3^neg^ T cells, consistent with impaired lineage stability when IL-2 signals are limiting. Thus, even if they can prevent autoimmunity in *Il2ra*^*mut/mut*^ mice, *Il2ra*^*mut/mut*^ T_reg_ cells may not be able to suppress WT autoreactive T_conv_ cells to the extent to which WT T_reg_ cells can.

**Fig. 7:**
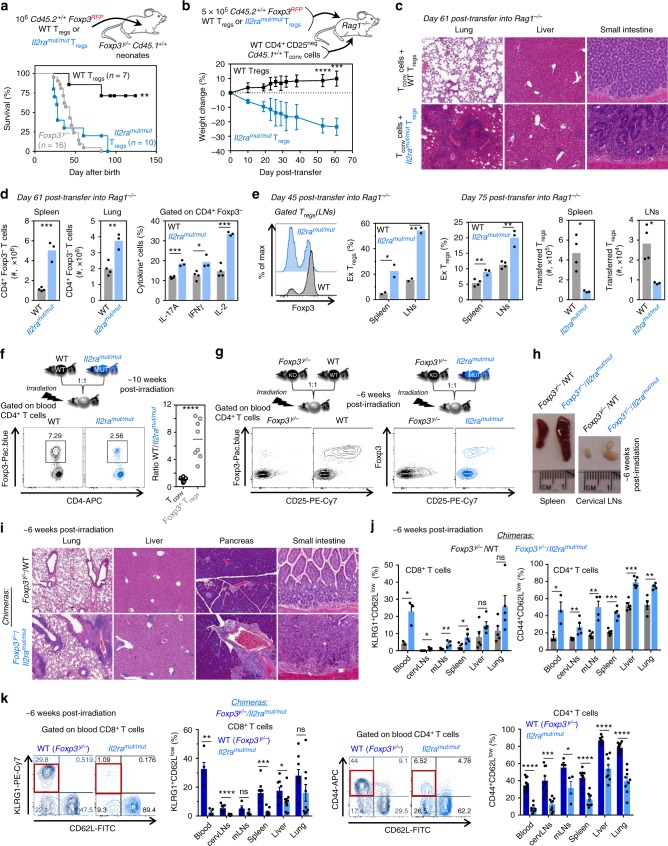
T_reg_ cells receiving limited IL-2 signals cannot suppress autoreactive WT T_conv_ cells: **a** *Foxp3*^*y/*−^ *Cd45.1*^*+/+*^ neonate mice were transferred i.p. with 10^6^ *Il2ra*^*mut/mut*^ or WT flow-purified *Foxp3*^*Rfp/Rfp*^ CD3^+^CD4^+^ T_reg_ cells and survival was monitored over 120 days. Graphs average results over multiple mice in each group and 7-16 replicate experiments. **b**, **c** *Rag1*^−*/*−^ mice received 5 × 10^5^ flow-purified *Foxp3*^*Rfp/Rfp*^ CD3^+^CD4^+^ T_reg_ cells together with 1.5 × 10^6^ WT CD3^+^CD4^+^CD25^neg^ T_conv_ cells i.v. Weight loss was monitored up to 61 days post transfer and immune cell infiltrates on H/E stained sections is shown at day 61 (*n* = 4). **d** Numbers of transferred *Foxp3*^*Rfp/Rfp*^ CD3^+^CD4^+^ T_reg_ cells in the spleen and lung of *Rag1*^−*/*−^ mice (left bar graphs) and proportion of cytokine-secreting CD3^+^CD4^+^ T_conv_ cells 4 h post PMA/ionomycin stimulation. **e** Foxp3 expression in *Foxp3*^*Rfp/Rfp*^ CD3^+^CD4^+^ T_reg_ cells 45 and 75 days post transfer, proportion and absolute numbers of ex-T_reg_ cells in LNs and spleens. **f**, **g** Schematic of mixed bone-marrow chimera generation in lethally irradiated WT B6 recipient mice with discriminative congenic markers. **f**, **g** Representative FACS dot plots of Foxp3 and CD4 expression in blood T cells of **f** WT*/Il2ra*^*mut/mut*^ and **g** *Foxp3*^*y/*−^/WT and *Foxp3*^*y/*−^*/Il2ra*^*mut/mut*^ chimeras 10 and 6 weeks post reconstitution, respectively. Graph in **f** shows the ratio of WT vs *Il2ra*^*mut/mut*^ T_conv_ and T_reg_ cells for each individual chimera. Data are across several chimeras (5–9 mice/group) in 3–4 replicate experiments. **h**, **i** Spleen, LN pictures, and H/E stained sections of lung, liver, pancreas, and small intestine from *Foxp3*^*y/*−^/WT and *Foxp3*^*y/*−^*/Il2ra*^*mut/mut*^ chimeras. **j** Cells from indicated organs from the two groups of chimeras were stained for cell-surface expression of CD3, CD4, CD8, CD62L, KLRG1, and CD44. Bar graphs show the proportion of activated CD4^+^ (CD44^+^CD62L^lo^) or CD8^+^ (KLRG1^+^CD62L^lo^) T cells (*n* = 5–9). **k** Proportion of activated CD3^+^CD4^+^ or CD8^+^ T_conv_ cells from *Il2ra*^*mut/mut*^ or WT BM in *Foxp3*^*y/*−^*/Il2ra*^*mut/mut*^ chimeras. Bar graphs summarize average proportion across mice (*n* = 5–9). p-values are indicated when relevant with *p < 0.05; **p < 0.01; ***p < 0.001; NS not significant, using two-tailed unpaired Student’s *t*-test. Symbols on bar graphs feature individual mice

To further evaluate their suppressive capacity in vivo, we used two distinct but complementary models of acute autoimmunity development, and a model of reconstitution at homeostasis. We first conducted rescue experiments of *Foxp3*^*y/*−^ mice^5^ which develop fatal autoimmunity within ~3–4 weeks post birth, by transferring T_reg_ cells sorted from the secondary lymphoid organs (SLOs) of WT or *Il2ra*^*mut/mut*^ *Foxp3*^*Rfp/Rfp*^ reporter mice to recipient neonates (day 1–3) (Fig. 7a). As expected, *Foxp3*^*y/*−^ mice receiving WT T_reg_ cells survived, while those transferred with *Il2ra*^*mut/mut*^ T_reg_ cells did not control the development of wasting disease and ultimately succumbed, as did untransferred counterparts. Second, we adoptively transferred CD4^+^CD25^neg^ T_conv_ cells to *Rag1*^−*/*−^ mice, which also results in a rapid wasting disease unless functional T_reg_ cells are co-transferred (Fig. 7b). As predicted from the first model, *Il2ra*^*mut/mut*^ T_reg_ cells fail to suppress autoreactive T_conv_ cell-mediated autoimmunity with a rapid loss of weight and massive infiltrates of T cells in multiple organs (lung, liver, small intestine) in contrast to control groups transferred with WT T_reg_ cells or only CD4^+^ T_conv_ cells (Fig. 7c and Supplementary Fig. 7c). Consequently, the absolute numbers of CD4^+^ T_conv_ cells were two (lung) to five (spleen) fold increased in autoimmune mice with a higher proportion secreting IL-2 (factor of 2), IL-17A, and IFNγ (Fig. 7d). Fate mapping of *Il2ra*^*mut/mut*^ or WT T_reg_ cells transferred to *Rag1*^−*/*−^ (Fig. 7e) or *Foxp3*^*y/*−^ (Supplementary Fig. 7d) mice further showed that Foxp3^+^ *Il2ra*^*mut/mut*^ T_reg_ cells become ex-T_reg_ cells and are lost compared to WT counterparts, both in proportion and absolute numbers. Thus, a threshold of IL-2 signals to T_reg_ cells is needed to program their functional attributes, for their maintenance, and to prevent wasting autoimmunity. Consistent with this conclusion and the lack of competitive fitness^21^ of *Il2ra*^*mut/mut*^ T_reg_ cells, a significantly lower proportion of *Il2ra*^*mut/mut*^ compared to WT T_reg_ cells was measured in WT B6 chimeric mice reconstituted with an equal ratio of *Il2ra*^*mut/mut*^ and WT bone-marrow donor cells while the proportion of CD4^+^ T_conv_ cells was maintained (Fig. 7f).

As a last step to assess *Il2ra*^*mut/mut*^ T_reg_ cells functional attributes, we created another chimeric mouse model at homeostasis in which T_reg_ cells lacked full IL-2 responsiveness while T_conv_ cells responded normally. We reconstituted lethally irradiated WT mice with bone marrow cells from *Foxp3*^*y/*−^ and *Il2ra*^*mut/mut*^ or control WT mice (Fig. 7g). In such *Foxp3*^*y/*−^/*Il2ra*^*mut/mut*^ chimeras, only T_reg_ cells lacked CD25 expression and mice exhibited enlarged spleens and LNs compared to the *Foxp3*^*y/*−^/WT control chimeras (Fig. 7h). Moreover, massive infiltrates of T cells were noted in lung, liver, pancreas and small intestine of *Foxp3*^*y/*−^/*Il2ra*^*mut/mut*^ mice, with gross architectural tissue alterations compared to the control group (Fig. 7i). In line with these findings, both CD4^+^ and CD8^+^ T cells in *Foxp3*^*y/*−^/*Il2ra*^*mut/mut*^ but not *Foxp3*^*y/*−^/WT chimeras underwent robust activation (CD62L^low^, KLRG1^+^, CD44^hi^, CD69^hi^) across multiple tissues (Fig. 7j and Supplementary Fig. 7e, f). Only WT but not *Il2ra*^*mut/mut*^ T_conv_ cells in *Foxp3*^*y/*−^/*Il2ra*^*mut/mut*^ chimeras were activated (Fig. 7k and Supplementary Fig. 7g), suggesting that equal responsiveness to IL-2 by T_conv_ cells and T_reg_ cells represent a major parameter safeguarding immune homeostasis. In summary, T_reg_ cells that can only integrate lower IL-2 signals than their T_conv_ cell counterpart, acquire a suboptimal epigenetic program, are less stable (Foxp3) and are impaired in their ability to deprive autoreactive T_conv_ cells of IL-2.

## Discussion

While the Foxp3 TF is required to maintain peripheral T_reg_ cell identity and functions, it is not sufficient to secure their stable epigenetic identity and program their functional features^12,16,24^. But how exactly are T_reg_ cell chromatin accessibility characteristics determined before Foxp3 is expressed is still hotly debated. TCR signaling represents the major cue driving thymic selection, and this plays a determining role in thymocytes commitment to the T_reg_ cell lineage^47^, however this is likely not sufficient^12,14,25^. Signals such as IL-2 and TGFβ cytokines, are suggested to contribute to the epigenetic modeling of T_reg_ cells^2,3^, yet current evidence for IL-2 mostly pointed to its role as a major stabilizer of Foxp3 expression. Here, our data support a role for IL-2 in defining the epigenetic identity of Foxp3^+^ T_reg_ cells, which appears largely independent of Foxp3. In fact, genome-wide chromatin accessibility changes when IL-2 signals are impaired are much wider than the regions where Foxp3 binds. Our whole genome analysis of OCRs in *Il2ra*^*mut/mut*^ vs WT T_reg_ cells from the LNs reveals that the epigenetic landscapes are widely distinct (~41%). Consistent with this result, constitutive STAT5 expression, which forces IL-2 signaling, is shown to divert thymocyte selection to the T_reg_ cell lineage^11^. Using data from a prior report on T_reg_ cell epigenetic signature under various conditions of activation^36^, we analyzed that ~36% of OCRs differed in activated vs resting Foxp3^+^ T_reg_ cells, a proportion comparable to the extent of IL-2-mediated remodeling observed in WT vs *IL2ra*^*mut/mut*^ LN T_reg_ cells. These differences are equivalent to what is documented between effector and memory or exhausted CD8^+^ T cells^38^, which are undergoing extensive chromatin remodeling upon differentiation^37–39^, overall supporting the conclusion that IL-2 signals significantly shape the epigenetic landscape of T_reg_ cells in vivo.

Because IL-2 signals mediate genome-wide chromatin remodeling beyond Foxp3, and already on mature Foxp3^+^ T_reg_ cells in the thymus, we postulated that this may occur in the thymus at the SP CD4^+^ thymocyte stage preceding Foxp3 expression in committed T_reg_ cells. The genome organizer SATB1, which is highly expressed during most stages of T cell thymic selection, was recently involved in setting-up thymic-derived T_reg_ cell functional features and ability to prevent autoimmunity before Foxp3 is turned on^27^. We further reveal that SATB1 positioning in the genome of SP CD4^+^ thymocytes impaired in IL-2 signaling, is much broader than in WT thymocytes, likely reflecting ectopic binding of this pioneer factor when IL-2 signals are limiting. Our finding that more than 20 times as many GO pathways are associated with the genes exhibiting unique OCRs in WT LN T_reg_ cells compared to *IL2ra*^*mut/mut*^ counterparts supports the idea that such ectopic binding of SATB1 lead to unfocused T_reg_ cell chromatin modeling. In WT thymocytes, SATB1 is more selectively bound to the TSS of genes encoding for TFs and nucleic acid binding proteins, which may in turn control the correct T_reg_ cell epigenetic landscape. Several TFs such as STAT5 and PI3/MAP kinase-dependent TFs, are activated downstream of IL-2 signaling. From our analysis of the SATB1 DNA binding regions and TFs known to bind to these motifs, we noted GATA3, RUNX1 and ELF1 that are all reported to be present prior to Foxp3 expression to enable its future recruitment and optimal function^15,17,26,43^. SATB1 is also described among the “quintet” TFs—together with Foxp3—that are essential for in vitro re-programing of T_conv_ cells to T_reg_ cells^16^. While still speculative, it is possible that IL-2 signals, in addition to TCR triggering, are essential to promote the expression/activation of the previous TFs and of SATB1 and/or other pioneer factors, to set-up the appropriate epigenetic landscape on which Foxp3 and these partner TFs act to achieve the final T_reg_ cell identity. Binding to the Foxp3 CNS3 and CNS2 elements for instance is essential to initiate and then stabilize expression of Foxp3^17,18,26,48^. The NF-kB subunit c-Rel binds CNS3, and STAT5 and GATA3^49^ stabilize CNS2. While we did not find evidence for SATB1-binding on c-Rel or STAT5 motifs, SATB1 may associate with GATA3 to help focus its binding to DNA area relevant to the acquisition of the T_reg_ cell signature prior Foxp3 is expressed. Such mechanism would prevent ectopic binding of SATB1 throughout the genome when IL-2 signals are limiting.

The consequence of lower IL-2 signaling in thymic-derived peripheral T_reg_ cells is a different epigenetic landscape, which we suggest is being set in the thymus through SATB1 together with other IL-2-dependent TFs. In the periphery, however, SATB1 is not required to sustain functional thymic-derived T_reg_ cells^27^. Some TF binding motifs found in SATB1-binding regions like RUNX1, which is likely to interact with SATB1 in SP CD4^+^ thymocytes, are also among the motifs found in the differential OCRs of WT compared to *Il2ra*^*mut/mut*^ T_reg_ cells in the periphery. RUNX1 binds to the IL-2 promoter and promotes its transcription, but is repressed by Foxp3 through physical interactions^42^, preventing the expression of an effector T cell program by T_reg_ cells. Other TF binding motifs only found in WT peripheral T_reg_ cells, such as FOXO1 and FOXO3, also bind to the Foxp3 promoter and conserved intronic enhancer regions, allowing for Foxp3 expression and T_reg_ cell-lineage specification^50^. IL-2-mediated induction of all these TFs at the pre-T_reg_ cell stage, via SATB1 or not, may help maintain the T_reg_ cell program in the periphery. Along these lines, we noted multiple differentially expressed TF-encoding genes (*Prdm1, Gata3, Dzip1, Irf4*), chromatin remodeling- and histone-encoding genes between WT and *Il2ra*^*mut/mut*^ LN T_reg_ cells.

Given that our data support a model in which IL-2 alters T_reg_ cell epigenetic identity early during thymic selection when thymocytes commit to this lineage, it is unlikely that this mechanism will be at work for induced T_reg_ cells in peripheral tissues^51^. Our analysis in WT vs *Il2ra*^*mut/mut*^ T_reg_ cells failed to reveal any differences in OCRs of genes encoding Foxp3 or HELIOS that were reported to exhibit differential methylation profiles in induced/peripheral T_reg_ cells^52^.

We report a role for IL-2 on Foxp3^+^ T_reg_ cells that is largely independent of Foxp3-mediated stabilization of T_reg_ cells and of their ability to capture IL-2 from activated T_conv_ cells. The Y129H mutation in CD25 prevents optimal folding and/or egress of CD25 to the surface of T cells, translating into decreased binding of IL-2 and subsequent signaling and proliferative response. Two closely comparable models were previously reported: in the first, mutations inside several of key signal transducing cytoplasmic tyrosines of the IL-2Rβ chain disrupted both IL-2 or IL-15 signaling^29^. The second model overexpressed a constitutively active form of the b subunit of the STAT5 TF^51^ on either IL-2Rβ (*Cd122*^−*/*−^)^19^ or IL-2Rγ (*γc*^−*/*−^) knockout backgrounds^11^. None of these models could differentiate between IL-2 and IL-15, or other γ-chain-dependent cytokine signaling, confounding interpretation on the roles of high affinity IL-2 signaling. Nevertheless, a rather intriguing finding in both of these settings, as in our model, is that receptor deficient mice did not develop autoimmunity, prompting the idea that T_reg_ cells were set to a low IL-2 receptor signaling threshold sufficient for Foxp3 induction and maintenance. Our current results, however, favor a different interpretation in which, when comparable IL-2 signals are received by T_reg_ and T_conv_ cells, the development of autoimmunity can be prevented. Yet, if T_reg_ cells cannot receive as much IL-2 signals as T_conv_ cells, mice will develop fatal wasting disease. A third model recently developed by Rudensky and colleagues, enabled to discriminate the contribution of IL-2 capture and T_reg_ cell stability in preventing fatal autoimmunity^40^. As T_reg_ cells cannot secrete IL-2^6^ but constitutively express the high affinity IL-2Rα/CD25 chain, a large conundrum in the field is their reliance on cell-extrinsic IL-2 from activated -potentially autoreactive- T_conv_ cells, thus preventing excessive T_conv_ cell proliferation and activation by physically capturing the IL-2 they secrete. This study brought strong evidence that IL-2 capture does represent a key mechanism of suppression used by T_reg_ cells. While the *Il2ra*^*mut/mut*^ mouse does not allow to assess the role of IL-2 capture by T_reg_ cells in maintaining immune homeostasis, the progression of disease and survival in *Foxp3*^*y/*−^ mice rescued with *Il2ra*^*mut/mut*^ T_reg_ cells, the *Rag*^−*/*−^ transfer experiments and the *Foxp3*^*y/*−^/*Il2ra*^*mut/mut*^ chimeras, are close to that observed in *Foxp3*^*y/*−^ mice. Length of survival is, however, distinct to that of the Rudensky model, in which mouse survival is approximately twice increased. In this latter model too, CD8^+^ but not CD4^+^ T cells, exhibit robust activation whereas in our *Foxp3*^*y/*−^/*Il2ra*^*mut/mut*^ chimeras, both subsets of T_conv_ cells are highly activated. Altogether, these observations support the idea that altering T_reg_ cell epigenetic identity, IL-2 capture and signaling leads to more rapid autoimmunity.

IL-2 therapy has been used for over two decades in patients, initially with the goal to boost anti-tumoral and HIV-specific immunity^52,53^. High doses of IL-2 were mostly used with important side effects and mitigated efficiencies. Yet the use of lower doses of IL-2, with the underlying rationale that T_reg_ cells would be more efficiently targeted than effector T cells, showed a preferential expansion of T_reg_ cells both in preclinical models and in type 1 diabetes patients, which was associated with better prognostic markers^54,55^. While the mechanism underlying these promising outcomes in patients may be accounted for by T_reg_ cell expansion, our results further raise the possibility that qualitative changes at the levels of chromatin accessibility and epigenetic reprogramming of T_reg_ cells during thymic selection, may contribute to ameliorate T_reg_ cell functions in autoimmune patients.

## Methods

### Ethics statement

This study was carried out in strict accordance with the recommendations by the animal use committees at the Albert Einstein College of Medicine. All efforts were made to minimize suffering and provide humane treatment to the animals included in the study.

### Mice

All mice were bred in our SPF animal facility at the Albert Einstein College of Medicine. We used wild-type (WT) C57BL/6J (B6) 6-8 weeks old mice, congenic CD45.1^+/+^ (#2014), *Ccr2*^−*/*−*2009*^ (#4999)*, Ccr2*^−*/*−*2012*^ (#4999)*, Foxp3*^*+/*−^ (#19933)*, Foxp3*^*Rfp/Rfp*^ (#8374)*, Rag1*^−*/*−^ (#2216) from the Jackson labs all on the B6 genetic background. *Ccr2*^*+/+2009*^ *Il2ra*^*mut/mut*^ (called *Il2ra*^*mut/mut*^*)* were generated by intercrossing to the B6. *Il15*^−*/*−^ (#4269) mice were purchased from Taconic.

### Microbial pathogens and mice infections

We used wild type *Listeria monocytogenes* expressing the Ovalbumin (Ova) model antigen (*Lm*-Ova) on the 10403s genetic background^56^. For infections, bacteria were grown to a logarithmic phase in broth heart infusion medium, diluted in PBS to infecting concentration (10^4^) and injected i.v. *For Herpes Simplex Virus 2 (HSV-2) infection*, female mice treated with 2 mg medroxyprogesterone acetate subcutaneously (s.c.) were immunized or not 5 days later intravaginally with 2 × 10^5^ plaque forming units (PFU) of 186ΔKpn HSV-2 (TK^−^ HSV-2) and organs (spleen and draining LNs) were harvested 7 days later.

### Cell suspensions for flow cytometry and adoptive transfers

Spleens, lymph nodes (inguinal and cervical), or thymuses were dissociated on nylon mesh while lungs, liver, and pancreas were cut. All organs were incubated in HBSS medium with 4000 U/mL collagenase I and 0.1 mg/mL DNase I, and red blood cells (RBC) lysed with NH_4_Cl buffer (0.83% vol/vol). Blood was harvested into heparin tubes and RBC lysed as before. Bone marrow cells were obtained from flushing femur with complete RPMI with 10% FCS.

### Cell-staining for FACS analysis

Cell suspensions were incubated with 2.4G2 Fc Block and stained with fluorescently tagged antibodies (Abs) purchased from eBioscience, BD Biosciences, Tonbo Bioscience, or BioLegend (Supplementary Table1) in PBS 1% FCS, 2 mM EDTA, 0.02% sodium azide. Biotinylated monomers (1 mg/mL) obtained from the NIH tetramer Core Facility, were conjugated with PE-labeled Streptavidin (1 mg/mL) as follow: 6.4 μL of PE-Streptavidin were added to 10 μL of monomers every 15 min four times on ice. Newly generated tetramers (1/400-1/500 dilution) were then used to stain spleen/LN cells for 1 h at 4 °C. For transcription factor (TF) intracellular staining (Foxp3, Blimp-1), cells were fixed in eBioscience Fixation/Permeabilization buffer prior to TF Ab staining in eBioscience Permeabilization buffer for 30 min. For intracellular cytokine staining (ICS), cells were incubated 4 h at 37 °C/5%CO_2_ in RPMI1640 10%FCS, Golgi Plug/Golgi Stop (BD), fixed in IC fixation buffer (eBioscience), and permeabilized prior to 30 min staining with Abs against indicated intracellular markers. For intracellular phosphorylated STAT5 staining, cells were starved in RPMI w/o FCS for 30 min before stimulation with variable concentration of recombinant human IL-2 (Gemini) for 20 min. Cells were then fixed and permeabilized with 4% PFA followed by 90% methanol, and stained with anti-pY-STAT5 antibody (BD Biosciences). Data acquisition was done using a BD LSR II or a FACS Aria III flow cytometer. All flow cytometry data were analyzed using FlowJo v9 software (TreeStar). Cell sorting of Foxp3^+^ and Foxp3^−^ cells was performed based on RFP expression in *Foxp3*^*Rfp/Rfp*^ reporter mice using a BD FACS Aria III cell sorter. The FACS gating strategies for the experiments shown and T cell sorting are provided in Supplementary Fig. 8.

### Transfection of wild-type and Y129H *Il2ra* in 293T cells

The *Il2ra* cDNA was amplified by PCR and cloned into pMSCV-IRES-GFP (pMIG, kind gift from Guy Sauvageau) to generate pMIG-*Il2ra*. PCR was performed on pMIG-*Il2ra* to introduce a point mutation on nucleotide 426 (thymidine to a cytosine), which results in a tyrosine-histidine conversion at amino-acid position 129. The *Il2ra*^*mut*^ cDNA was then cloned into pMIG to generate pMIG-*Il2ra*^*mut*^. The presence of the introduced point mutation was confirmed by sequencing.

293T cells were plated 2 days before transfection on 100 mm adherent petri dish at 10^6^ cells per plates. Cells at 70% confluence were transfected with 30 µg of the plasmid pMIG, pMIG-*Il2ra* or pMIG-*Il2ra*^*mut*^ using 45 µl Lipofectamine 2000 in 4.5 mL OptiMEM (ThermoFisher) overnight in antibiotic-free medium. Medium was replaced the day after transfection and 3 days later, cells were trypsinized and analyzed for CD25 extra- and intracellular expression by flow cytometry as described before. The 293T cells were a kind gift from Heather Melichar (University of Montreal, Canada) and were tested mycoplasma negative.

### In vitro T_conv_ and T_reg_ cell assays

For all assays, naive CD4^+^ or CD8^+^ T_conv_ cells were negatively selected from spleen and LNs using either anti-CD8β (H35) or anti-CD4 (GK1.5), anti-CD11b (M1/70), anti-MHC-II (M5/114), anti-TER119, anti-B220 (RA3-6B2) and anti-CD25 (PC61), all at 5 μg/mL for 30 min at 4 °C. Cells were then washed and incubated with anti-rat magnetic beads at 1 bead/target cell for 30 min at 4 °C (Dynabeads sheep anti-rat IgG, Invitrogen). For suppression assays, purified naive WT *Cd45.1*^*+/+*^ CD4^+^ T_conv_ cells were stained with 1–5 μM of CellTrace Violet (CTV, Invitrogen) according to the manufacturer’s protocol. WT and *Il2ra*^*mut/mut*^ *Foxp3*^*Rfp/Rfp*^ T_reg_ cells were sorted by flow cytometry (Aria III). 5 × 10^4^ CTV-labeled naive CD4^+^ T_conv_ cells were cultured with increasing numbers of T_reg_ cells in the presence of 10^5^ irradiated, T cell-depleted WT B6 splenocytes and 1 μg/mL anti-CD3ε (clone 145-2C11, BD) in a 96 round-bottom plate for 72 h. Cell proliferation of T_conv_ cells (live CFSE^−^CD4^+^Foxp3^−^) was determined by flow cytometry based on CTV fluorescence intensity dilution of T_conv_ cells. For proliferation assays, CTV-labeled purified WT and *Il2ra*^*mut/mut*^ naive CD4^+^ or CD8^+^ T_conv_ cells were incubated for 24 h on anti-CD3ε (10 μg/mL) pre-coated wells before co-culture for 96 h with varied concentrations (2.5-10^4^ U/mL) of human recombinant IL-2 (Gemini Bio-product). Cell proliferation was determined by flow cytometry and CTV fluorescence dilution of T_conv_ cells. For short-term activation assays for immunofluorescence, purified WT and *Il2ra*^*mut/mut*^ naive CD4^+^ T cells were incubated for 48 h with anti-CD3ε (10 μg/mL) pre-coated wells to induce CD25 upregulation. At 48 h, cells were transferred in polylysine pre-coated chambers, left to adhere for 1 h at 37 °C, fixed with 1% PFA and permeabilized with 0.1% Triton X-100 prior to OVN staining in PBS 0.5% BSA, 0.05% Triton X-100 containing polyclonal goat anti-mouse CD25 (RD systems) and rabbit anti-calreticulin Abs (1/300 dilution, ThermoFisher, PA3-900). Staining was revealed by staining for 2 h with secondary anti-goat-Alexa 488 and anti-rabbit-Alexa 546 Abs (Invitrogen). Cells were then covered with Fluoromount-G (SouthernBiotech) and imaged using a Zeiss Axiovert microscope (Carl Zeiss Microimaging Inc., Thornwood, NY) with a ×63 NA 1.4 objective and a Retiga 2000 camera. Green channel images using a 450–490 excitation/500–550 emission bandpass filter and red staining was imaged with a 565/30 excitation-620/60 emission bandpass filter. Images were processed using Adobe Photoshop CS 4 (Adobe Systems, Inc. San Jose, CA).

### In vivo T_reg_ cell functional assays

For mixed bone-marrow chimera mice*, Rag1*^−*/*−^*Cd45.2*^*+/+*^ mice were lethally irradiated with 1200 rads before immediate reconstitution with 5 × 10^6^ T cell-depleted (as for the in vitro assays) bone marrow from *Foxp3*^−*/y*^*Cd45.1*^*+/+*^ and *Il2ra*^*mut/mut*^*Cd45.1*^*+/*−^ or congenic *Cd45.1*^*+/+*^ and *Il2ra*^*mut/mut*^*Cd45.1*^*+/*−^ at a 1:1 ratio. Mice were placed under antibiotics for 2 weeks and reconstitution ratios were checked by FACS 4–6 weeks later. In some experiments, C57BL/6 *Cd45.2*^*+/+*^ were used as recipient. In such case, mice received 150 μg of anti-CD8β and anti-CD4 i.v. with for two consecutive days to deplete T cells prior irradiation.

For Rag1^−/−^ T cell transfers, LN- and spleen-purified naive CD4^+^ T_conv_ cells (by negative selection as above) isolated from Cd45.1^+/+^ male mice were transferred to Rag1^−/−^ recipients alone or mixed at a 5:1 ratio with flow-sorted sorted (Aria III) RFP^+^ T_reg_ cells from spleen and LNs of WT or *Il2ra*^*mut/mut*^ *Foxp3*^*Rfp/Rfp*^ reporter mice (2.5 × 10^6^ cells per recipient). In some experiments (when indicated), naive CD4^+^ T cells were labeled with 10 nM of CTV prior adoptive transfer. Recipient mice were monitored for body weight changes and transferred lymphocyte subsets were monitored by flow cytometry 45 and 75 days later.

For *Foxp3*^−*/y*^ mice rescue experiments, 10^6^ flow-sorted T_reg_ cells from WT or *Il2ra*^*mut/mut*^ *Foxp3*^*Rfp/Rfp*^ reporter mice were injected i.p. into 1–2-day-old pups (*Foxp3*^*y/−*^ males, genotyped after birth) from females *Foxp3*^*+/*−^ mice bred with *Cd45.1*^*+/+*^ congenic males. Male pups were assessed for the development of lymphoproliferative syndrome in blood and LNs at ~30 days of age.

For the antibody neutralization treatment, mice were given 150 µg of anti-ICOSL (clone HK5.3, BioXcell) by i.v. injection on days 0, 3, 6, 9, and 12 and sacrificed on day 14.

### Histological analysis

Tissue samples were fixed in 10% neutral buffered formalin and processed for hematoxylin and eosin staining. For each organ collected and for each genotype, two sections were cuts at 100 μm apart and all slides were scanned using a P250 High Capacity Slide Scanner (Perkin Elmer).

### Microarrays

Overall, 50,000 RFP^+^ T_reg_ or naive (CD62L^hi^CD44^lo^) RFP^neg^CD25^neg^CD4^+^ T_conv_ cells from LNs of WT or *Il2ra*^*mut/mut*^ *Foxp3*^*Rfp/Rfp*^ mice were flow-sorted based on the RFP signal and indicated markers after enrichment for CD4^+^ T cells (using negative selection, as described above). Pelleted cells were stored in 700 μL of TRIzol (Life Technologies) at −80 °C until RNA extraction. Total RNA was extracted using the RNAeasy Micro kit with RNase-Free DNase Set (Qiagen) according to the manufacturer protocol. The quality score and quantity of purified RNA was assessed with a Bioanalyzer RNA 6000 Pico Chip (Agilent). Total RNA was then converted to cDNA, amplified and hybridized to Affymetrix Mouse Transcriptome Array 1.0 Pico. Raw CEL files were preprocessed and normalized using Affymetrix Expression Console (version 1.4.1.46) and resulting data were analyzed with the Affymetrix Transcriptome Analysis Console (version 3.1.0.5). We calculated fold-differences between experimental groups and tested significance using one-way ANOVA (unpaired). Significantly up and downregulated genes were defined with at least a 1.5-fold expression difference and a *p*-value ≤ 0.01. Over-representation of biological-process (BP) gene-ontology (GO) terms was calculated using BiNGO (version 3.0.3) in Cytoscape (version v3.4.0), employing the hypergeometric test and applying a significance cutoff of FDR-adjusted *p*-value ≤ 0.05. The GO ontology and annotation files used were downloaded on May. 25, 2017. The output of BiNGO was imported into EnrichmentMap (version 2.1.0) in Cytoscape to cluster redundant GO terms and visualize the results. An EnrichmentMap was generated using a Jaccard similarity coefficient cutoff of 0.4, a *p*-value cutoff of 0.001, an FDR-adjusted cutoff of 0.005, and excluding gene sets with fewer than 5 genes. The network was visualized using a perfuse force-directed layout with default settings and 500 iterations. Groups of similar GO terms were manually circled. Finally, for analysis of gene expression in thymocyte fractions shown in Supplementary Fig. 5c, raw data from the NCBI database (GEO GSE15907) were analyzed^41^.

### Epigenetic profiling

For ATAC-seq experiments, we performed the analysis on two (LNs) or four (thymus) biological replicates per group as previously described^35^. Briefly, nuclei were isolated from 50,000 CD4^+^ T cell-enriched (by negative selection) flow-sorted RFP^+^ T_reg_ cells from WT or *Il2ra*^*mut/mut*^ *Foxp3*^*Rfp/Rfp*^ mice (LNs or thymus) using a solution of 10 mM Tris-HCl, 10 mM NaCl, 3 mM MgCl_2_, and 0.1% IGEPAL CA-630. Immediately following nuclei isolation, the transposition reaction was conducted using Tn5 transposase and TD buffer (Illumina) for 45 min at 37 °C. Transposed DNA fragments were purified using Qiagen Mini-Elute Kit and PCR amplified using NEB Next High Fidelity 2× PCR master mix (New England Labs) with dual indexes primers (Illumina Nextera).

For SATB1 ChIP-seq experiments, we performed the ChIP-seq following the Mayers Lab ChIP-seq Protocol v011014 which is one of the suggested protocols by ENCODE project with slight modifications (https://www.encodeproject.org/documents/6ecd8240-a351-479b-9de6-f09ca3702ac3/@@download/attachment/ChIP-seq_Protocol_v011014.pdf). Single positive (SP) CD4^+^ thymocytes from thymus of WT and *Il2ra*^*mut/mut*^ mice on two biological replicates were pre-enriched by negative selection using anti-CD8β (H35), anti-MHC-II (M5/114), anti-CD11b (M1/70) and anti-TER119. After staining the remaining cells with fluorescent labeled antibodies against CD4 (GK1.5) and CD8α (53-6.7), we flow-sorted 2 × 10^6^ CD4^+^ CD8α^neg^ SP thymocytes. Sorted cells were next cross-linked in 1% (wt/vol) formaldehyde solution for 30 min. Cross-linked DNA was lysed in Farnham lysis buffer (5 mM PIPES pH 8.0, 85 mM KCl and 0.5% NP-40), fragmented in RIPA buffer (1% NP-40, 0.5% sodium deoxycholate, and 0.1% SDS in 1×PBS) using Bioruptor (Diagenode), and incubated overnight at 4 °C with 100 µL of DynaBeads M-280 Sheep anti-Rabbit IgG magnet beads (Invitrogen) preincubated with 20 µg of anti-SATB1 antibody (Abcam, ab70004). Beads were washed five times with LiCl wash buffer (100 mM Tris pH 7.5, 500 mM LiCl, 1% NP-40 and 1% sodium deoxycholate) and one time with 10 mM Tris/0.1 M EDTA. SATB1-bound DNA was then eluted from the beads and reverse cross-linked by incubating the beads pellet in 200 µl of IP elution buffer (1% SDS and 0.1 M NaHCO_3_) at 65 °C overnight and further purified with MinElute PCR Purification Kit (QIAGEN). The library was prepared using Accel-NGS 2 S Plus DNA library kit according to the manufacturer’s instructions.

### Sequencing libraries processing

The size distribution and molarity of the sequencing library were determined by using an Bioanalyzer analysis (High Sensitivity DNA chip, Agilent). Sequencing was performed using a HiSeq 2500 system (Illumina). Obtained sequences were mapped to the mouse mm10 reference genome using BWA mem^57,58^. After eliminating duplicated reads and the reads aligned to mitochondrial DNA, we kept only concordantly mapped pairs for further analysis. Peak calling was performed on shifted reads using MACS v2.1 with narrow peaks option to identify areas of sequence tag enrichment following the original report^35^. For ATAC-seq, read1 reads were shifted using bedtools2, as previously performed in Buenestro et al.^35^, before calling peaks for each replicate. We performed Irreproducible Discovery Rate analysis for finding reproducible peaks among the biological replicates (0.05 cutoff)^59^. Analysis was performed in R (the R project) using Bioconductor packages. The genomic locations, overlapping or nearest genes and finding overlapping peaks between groups were annotated with ChIPpeakAnno^60^ and ChIPseeker^61^ using TxDb.Mmusculus.UCSC.mm10.knownGene annotation (Bioconductor). The motif analysis was performed on the genomic sequences of peaks using MEME-ChIP ^63^ and HOMER^62^. Over-representation of biological-process BP-GO terms was calculated using BiNGO as described above. For the ATAC-seq data analysis, the output of BiNGO was further filtered by selecting GO with an FDR-adjusted cutoff < 0.001 and excluding gene sets with fewer than five genes. The list of BP-GO was then imported into REViGO using a similarity coefficient of 0.7 and the SimRel columns to generate semantic similarities scores. The scored terms were visualized in semantic similarity-based scatterplots. For the SATB1 ChIP-seq data analysis, the output of BiNGO was imported into EnrichmentMap (version 2.1.0) in Cytoscape to cluster redundant GO terms and visualize the results as described above.

### Statistics

Statistical significance was calculated using an unpaired Student t test with GraphPad Prism software and two-tailed P values are given as: (*) *p* < 0.1; (**) *p* < 0.01; (***) *p* < 0.001; (****) *p* < 0.0001 and (ns) *p* > 0.1. All *p* values of 0.05 or less were considered significant and are referred to as such in the text. Error bars are mean ± SEM in all figures.

## Supplementary information

Supplementary Information

Peer Review File

Description of Additional Supplementary Files

Supplementary Data 1

Supplementary Data 2

Supplementary Data 3

Supplementary Data 4

Supplementary Data 5

Supplementary Data 6

Supplementary Data 7

Supplementary Data 8

Source Data

## Data Availability

The accession number for microarrays, ATAC-seq, and ChIP-seq data reported in this paper underlying Figs. 3–5 and Supplementary Figs 3–5 is GEO: GSE103217. The authors declare that all other data supporting the findings of this study are available within the paper and its supplementary information files. A reporting summary Article is available as a Supplementary Information file. The source data underlying Figs. 1a, c, d, g, h, 2a–e, 6a, c–f and 7a, b, d, e, j, k and Supplementary Figs 1b, d, 2b, d, 4c, 6b-d, f and 7a, b, f, g are provided as a Source Data file.
