# Supplementary material for: Interleukin 2 modulates thymic-derived regulatory T cell epigenetic landscape
Source: Nat Commun. 2018 Dec 18;9:5368. doi: 10.1038/s41467-018-07806-6 (PMC6299086; doi:10.1038/s41467-018-07806-6)
Supplement: Supplementary file 1 — Supplementary Information [file 41467_2018_7806_MOESM1_ESM.pdf]

Supplementary information for

Interleukin 2 modulates thymic-derived regulatory T cell epigenetic landscape

Laurent Chorro[1], Masako Suzuki[2], Shu Shien Chin[1], Tere M. Williams[1], Erik L. Snapp[3+], Livia Odagiu[4], Nathalie Labrecque[4] and Grégoire Lauvau[1*]

[1]Albert Einstein College of Medicine, Department of Microbiology and Immunology, 1301 Morris Park Avenue, Bronx,
NY, USA, 10461

[*]Correspondence should be addressed to GL: Albert Einstein College of Medicine, Department of Microbiology and Immunology, 1301 Morris Park Avenue, Price Center, Bronx, NY, 10461, USA. Ph: +1 718 678 1188, Fax: +1 718 678 1085, E-mail: gregoire.lauvau@einstein.yu.edu

Supplementary Figures

Supplementary Figure 1| A single substitution in a conserved tyrosine of IL-2Rα/CD25 accounts for impaired cell surface CD25.

Supplementary Figure 2| Functional characterization of the CD25 Y129H mutation.

Supplementary Figure 3| Epigenetic landscape of thymic and LN *Il2ra*$^{mut/mut}$ versus WT T_{reg} cells.

Supplementary Figure 4| Gene expression program of *Il2ra*$^{mut/mut}$ versus WT T_{reg} cells:

Supplementary Figure 5| Foxp3 binding does not account for IL-2-mediated epigenetic changes.

Supplementary Figure 6| Characterization of *Il2ra*$^{mut/mut}$ mice.

Supplementary Figure 7| Function and stability of *Il2ra*$^{mut/mut}$ T_{reg} cells.

Supplementary Figure 8|. FACS sequential gating and sorting strategies used in the study.

Supplementary Table

Supplementary Table 1| List of mAbs used in the study.

Supplementary Data Files

Supplementary Data File 1 (related to Fig. 3b)| List of common and unique ATAC-seq peaks in WT and *Il2ra*$^{mut/mut}$ thymic and LN T_{reg} cells across gene organization.

Supplementary Data File 2 (related to Fig. 3d)| List of genes with unique peaks in WT versus *Il2ra*$^{mut/mut}$ thymic and LN T_{reg} cells.

Supplementary Data File 3 (related to Fig. 3e and Sup Fig. 3d)| List of Biological Process Gene Ontology (BP-GO) from genes with unique peaks (OCRs) in WT versus *Il2ra*$^{mut/mut}$ thymic and LN T_{reg} cells (all and 20kb from TSS).

Supplementary Data File 4 (related to Fig. 4c)| List of genes up and downregulated in WT versus *Il2ra*$^{mut/mut}$ T_{reg} cells.

Supplementary Data File 5 (related to Fig. 4d and Sup Fig. 4d,e)| List of Biological Process Gene Ontology (BP-GO) from genes upregulated in *Il2ra*$^{mut/mut}$ versus WT T_{reg} cells.

Supplementary Data File 6 (related to Fig. 4e,g)| List of differentially expressed genes in WT versus *Il2ra*$^{mut/mut}$ LN T_{reg} cells compared to genes with unique peaks in WT and *Il2ra*$^{mut/mut}$ T_{reg} cells within 500 bp from TSS.

Supplementary Data File 7 (related to Fig. 5d)| List of unique genes with (i) SATB1 peaks in WT SP $CD4^+$ thymocytes, (ii) SATB1 peaks in *Il2ra*$^{mut/mut}$ SP $CD4^+$ thymocytes and (iii) common genes with unique SATB1 peaks in WT and *Il2ra*$^{mut/mut}$ SP $CD4^+$ thymocytes.

Supplementary Data File 8 (related to Fig. 5e,f)| BP-GO terms of unique genes with SATB1 peaks in WT and in *Il2ra*$^{mut/mut}$ SP $CD4^+$ thymocytes.

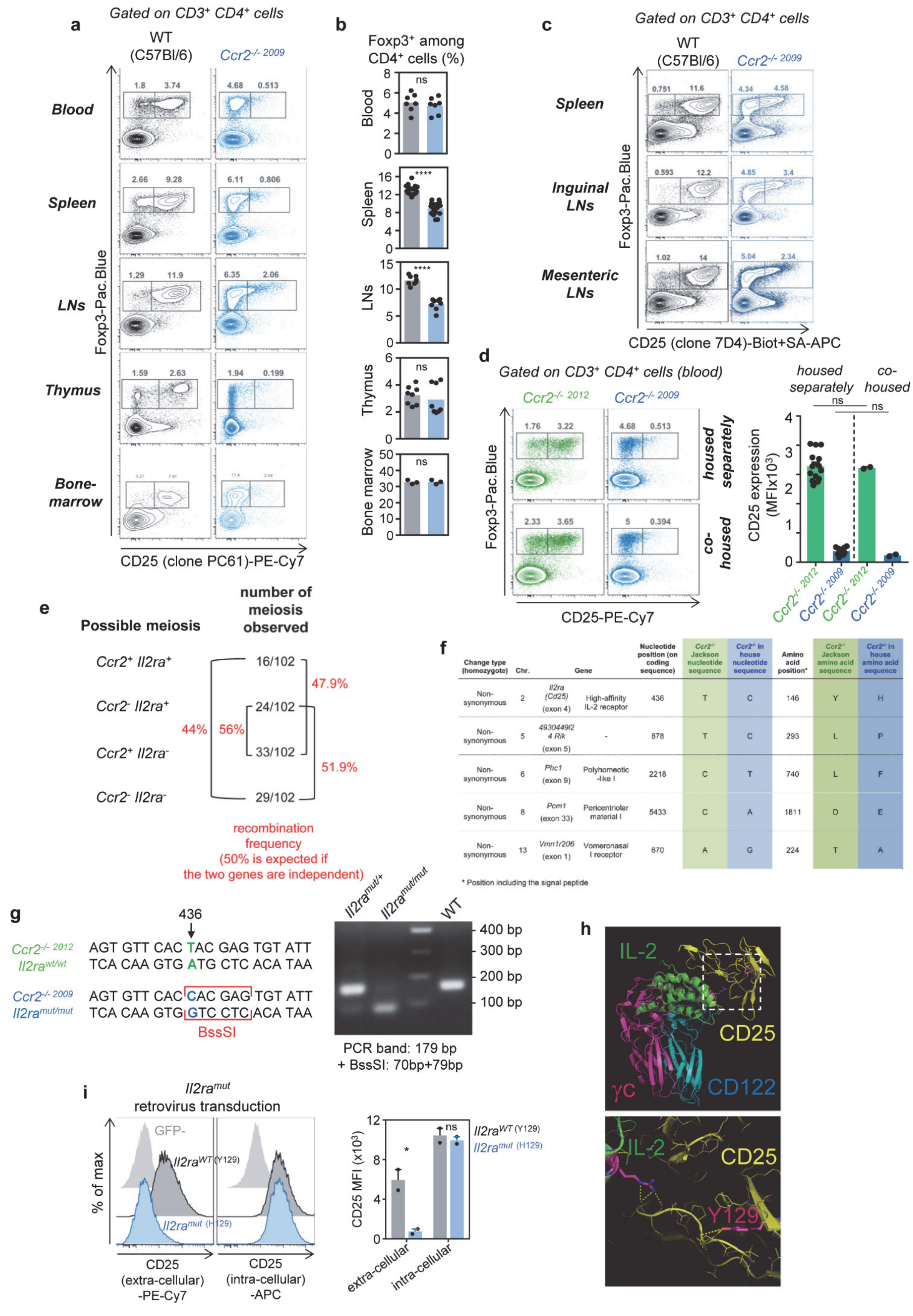

Change type (homozygote)	Chr.	Gene		Nucleotide position (on coding sequence)	Ccr2-/- Jackson nucleotide sequence	Ccr2-/- in house nucleotide sequence	Amino acid position*	Ccr2-/- Jackson amino acid sequence	Ccr2-/- in house amino acid sequence
Non-synonymous	2	*Il2ra (Cd25)* (exon 4)	High-affinity IL-2 receptor	436	T	C	146	Y	H
Non-synonymous	5	*4930449I24 Rik* (exon 5)	-	878	T	C	293	L	P
Non-synonymous	6	*Phc1* (exon 9)	Polyhomeotic-like I	2218	C	T	740	L	F
Non-synonymous	8	*Pcm1* (exon 33)	Pericentriolar material I	5433	C	A	1811	D	E
Non-synonymous	13	*Vmn1r206* (exon 1)	Vomeronasal I receptor	670	A	G	224	T	A

* Position including the signal peptide

Supplementary Figure 1| A single substitution in a conserved tyrosine of IL-2Rα/CD25 accounts for impaired cell surface CD25. (**a**) Cells from indicated organs of WT or *Ccr2*$^{-/-2009}$ (all on C57BL/6J background) mice were stained with mAbs against cell-surface CD3, CD4, CD25 (clone PC61) and intracellular Foxp3. Representative FACS dot plots are shown. (**b**) Bar graphs average % Foxp3^{+} T_{reg} cells among total CD3^{+}CD4^{+} T cells in indicated organs. (**c**) Cells from indicated organs of WT or *Ccr2*$^{-/-2009}$ mice were isolated and stained with mAbs against cell-surface CD3, CD4 and a distinct clone against CD25 (7D4) and intracellular Foxp3. Representative FACS dot plots are shown. (**d**) *Ccr2*$^{-/-2009}$ and *Ccr2*$^{-/-2012}$ mice were either housed separately or co-housed for 3 months prior to staining peripheral blood cells as before. A representative FACS dot plot of CD25 and Foxp3 expression in CD3^{+}CD4^{+} T cells is provided with bar graph showing the average cell-surface expression of CD25 expression (MFI) on Foxp3^{+} T cells (n=4 mice). (**e**) Number of meiosis enumerated in the *Ccr2*$^{+/-2009}$ x *Ccr2*$^{+/-2009}$ F2 offspring mice. (**f**) Whole exome sequencing was performed on genomic DNA of *Ccr2*$^{-/-2009}$ and *Ccr2*$^{-/- 2012}$ mice. The table show the five homozygote non-synonymous mutations found in *Ccr2*$^{-/-2009}$ compared to *Ccr2*$^{-/-2012}$ mice. For each mutation, the table shows the gene name, chromosome position, the switched nucleotide, the corresponding amino acid position and the switched amino acid. (**g**) DNA sequence alignments of the mutation found in exon 4 of *Il2ra* gene in *Ccr2*$^{-/-2009}$ versus *Ccr2*$^{-/-2012}$ mice. The mutation of a Cytosine by a Thymidine on position 149 in *Ccr2*$^{-/-2009}$ mice creates a new restriction site for the enzyme BssSI. To genotype mutant mice, a PCR was designed with primers flanking the mutation (Forward: 5'GAGCCACCTCCTTGGAAACA3' and Reverse: 5'CCCGTTTTCCCACACTTCAT3') creating a fragment of 179 bp. The PCR products was further digested with BssSI to create fragments of 70 and 79 bp. An agarose gel picture shows the migration of such PCR products after BssSI digestion from DNA of *Ccr2*$^{-/-2012}$ (WT), *Il2ra*$^{mut/+}$ and *Il2ra*$^{mut/mut}$ mice. (**h**) The human structure of IL-2/IL-2R complex is known. Y129 is conserved between human and mice as well as surrounding amino-acids. The close-up picture (swiss-model software) on the left shows the position of Y129 within CD25 protein (yellow). This Y (purple) is potentially involved in making hydrogen bounds (dotted lines) with the chain of CD25 that bind IL-2 (green). (**i**) FACS histograms of cell surface or intracellular expression of CD25 in 293 T cells retrovirally transduced with WT or mutagenized Y129H *Il2ra* cDNA. Bar graph shows CD25

expression (MFI) across 2 independent transduction experiments. P-values are indicated when relevant with *$p < 0.05$; **$p < 0.01$; ***$p < 0.001$; NS, not significant, using two-tailed unpaired Student's t test.

a

	Kd / apparent WT	Kd / apparent *Il2ra^mut/mut^*	Ratio
IL-2 binding (T_{conv} cells)	3.8	0.6	~6
STAT5 phosphorylation			
LN T_{reg} cells	12.5	0.725	~17
Thymic T_{reg} cells	15.3	0.79	~19
$CD4^+$ T_{conv} cells	68.4	1.69	~40
T cell proliferation	310.8 (CD8) / 446.4 (CD4)	3.9 (CD8) / 8.9 (CD4)	~78 (CD8) / 50 (CD4)

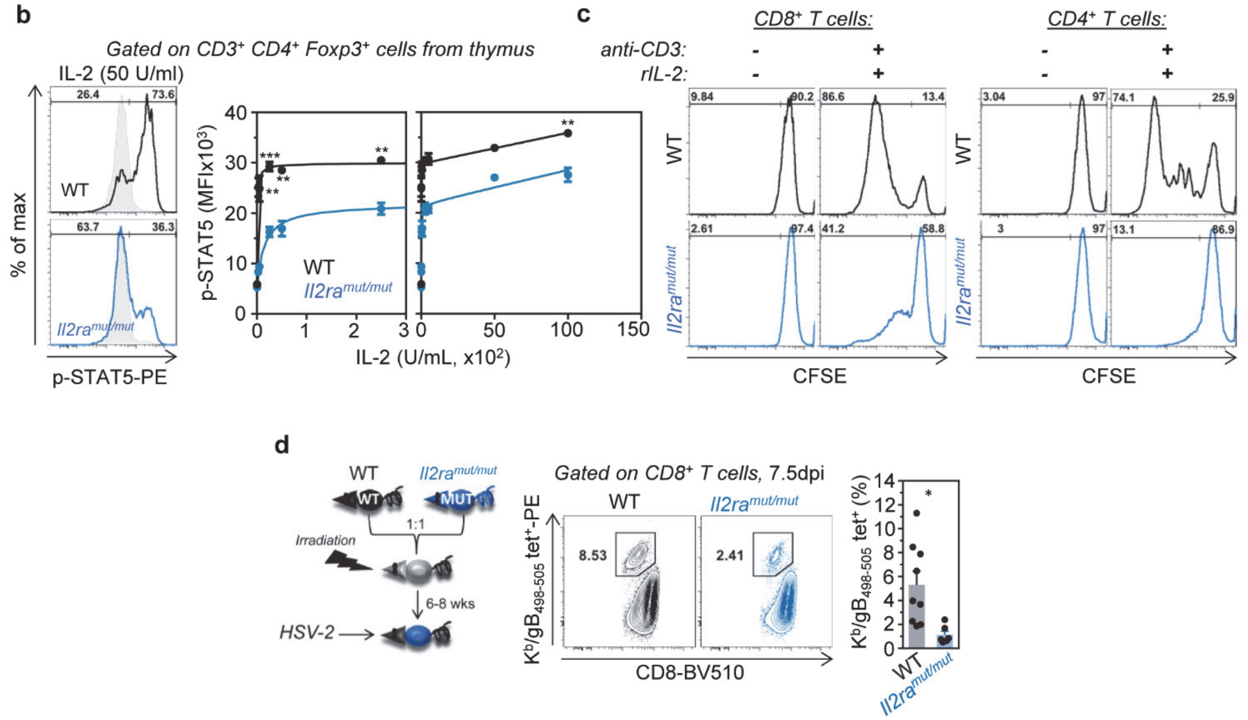

Supplementary Figure 2| Functional characterization of the CD25 Y129H mutation: (**a**) Summary of all measurements: IL-2 binding, IL-2 early signal transduction (STAT5 phosphorylation), and IL-2 induced T cell proliferation (CFSE) in *Il2ra^mut/mut^* and WT T_{conv} cells. (**b**) Thymocytes from *Il2ra^mut/mut^* or WT mice were incubated in serum free media (30') and increasing amount of IL-2 was added for 20' before proceeding to intracellular STAT5 phosphorylation staining. Representative FACS histograms of STAT5 phosphorylation levels in $Foxp3^+$ T_{reg} cells are shown. Graph represents phosphorylated STAT5 expression levels with increase IL-2. (**c**) Representative FACS dot plots of CFSE dilution of anti-CD3 stimulated T_{conv} cells from Figure 3c. (**d**) WT/*Il2ra^mut/mut^* mixed bone marrow (BM) chimeras (ratio 1:1) were immunized i.v. with the Herpes Simplex Virus 2 (HSV2, $2x10^5$ PFUs) and 7.5 days later spleen cells were stained with $gB_{498-505}/K^d$ tetramers. Representative FACS dot plots and summary bar graphs of HSV2-specific *Il2ra^mut/mut^* or WT $CD8^+$ T cells are shown (n=7 mice). P-values are

indicated when relevant with *$p < 0.05$; **$p < 0.01$; ***$p < 0.001$; NS, not significant, using two-tailed unpaired Student's t test.

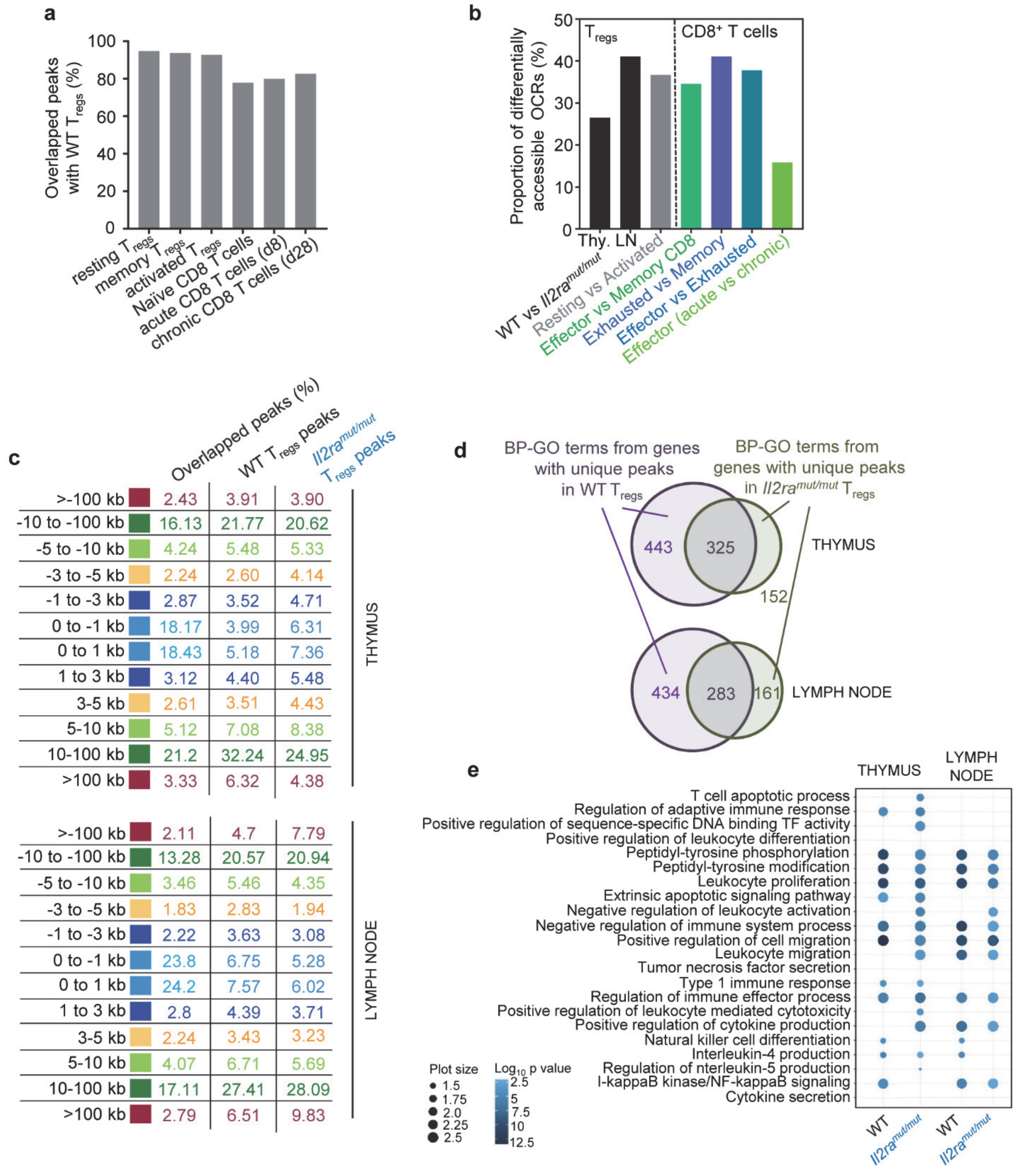

Supplementary Figure 3| Epigenetic landscape of thymic and LN *Il2ra*$^{mut/mut}$ versus WT T$_{reg}$ cells. T$_{reg}$ cells (5x10^{4}) were sorted by flow cytometry from thymus and LNs of *Il2ra*$^{mut/mut}$ and WT *Foxp3*$^{Rfp/Rfp}$ reporter mice, lysed and DNA from nucleus extracted for analysis of open chromatic regions by ATAC-seq. **(a)** Percent overlap in OCRs (peaks) in WT LN T$_{reg}$ cells from our study compared to (i) T$_{reg}$ cells (resting, memory, activated) reported by van der Veeken et al.

[1], and (ii) to CD8$^+$ T cells (naïve, effector or exhausted) reported by Scott-Browne et al. [2] . Raw data obtained from these reports were processed using the same bioinformatics tools as described for our own ATAC-seq analyses. **(b)** Number of differentially accessible peaks between WT and *Il2ra$^{mut/mut}$* thymic and LN T_{reg} cells from our study compared to (i) resting *vs.* activated T_{reg} cells using raw data generated by van den Veeken et al. [1] in our pipeline and (ii) effector *vs.* memory, exhausted *vs* memory or effector *vs.* exhausted CD8$^+$ T cells after LCMV infection reported by Sen et al. [3]. **(c)** Actual values for representation shown in Figure 3c. **(d)** Venn diagrams of number of GO pathways exhibiting unique peaks in *Il2ra$^{mut/mut}$* versus WT thymic and LN T_{reg} cells with no limit from TSS. **(e)** Network analysis of biological-process GO term enrichment among genes with unique peaks with no limit from TSS in *Il2ra$^{mut/mut}$* or WT thymic and LN T_{reg} cells. Genes were analyzed for over-represented GO terms using ReviGO. Node color is proportional to the FDR-adjusted P-value of the enrichment. Node size is proportional to gene set size.

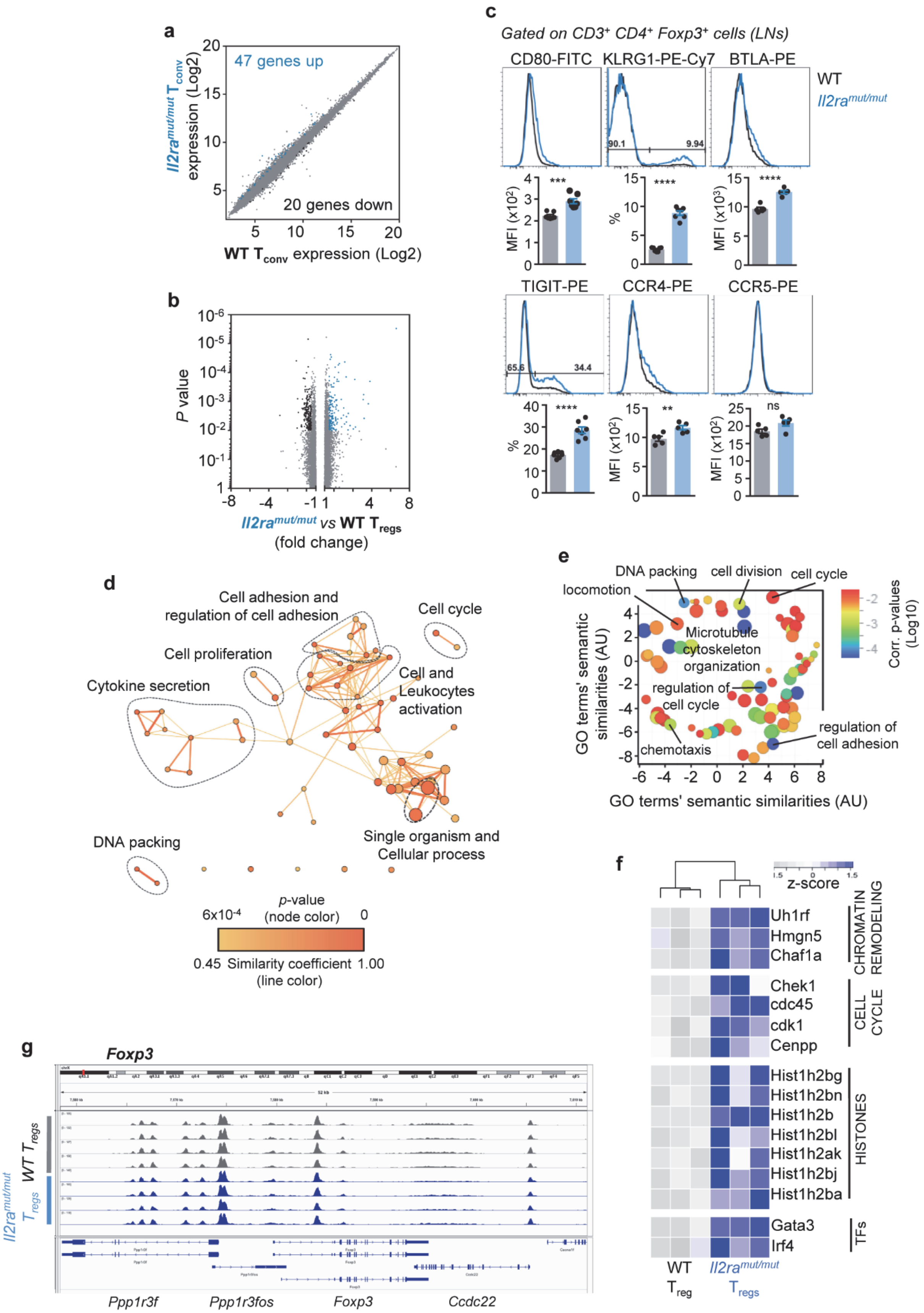
a
47 genes up
20 genes down
Il2ra^mut/mut T_conv expression (Log2)
WT T_conv expression (Log2)
b
P value
Il2ra^mut/mut vs WT T_regs
(fold change)
c
Gated on CD3+ CD4+ Foxp3+ cells (LNs)
CD80-FITC
KLRG1-PE-Cy7
BTLA-PE
WT
Il2ra^mut/mut
90.1
9.94
MFI (x10²)
%
MFI (x10³)
***
****
****
TIGIT-PE
CCR4-PE
CCR5-PE
65.6
34.4
**
ns
d
Cell adhesion and
regulation of cell adhesion
Cell cycle
Cell proliferation
Cytokine secretion
Cell and
Leukocytes
activation
DNA packing
Single organism and
Cellular process
6x10-4
p-value
(node color)
0
0.45 Similarity coefficient 1.00
(line color)
e
DNA packing
cell division
cell cycle
locomotion
Microtubule
cytoskeleton
organization
regulation of
cell cycle
regulation of
cell adhesion
chemotaxis
GO terms' semantic similarities (AU)
Corr. p-values
(Log10)
f
z-score
Uh1rf
Hmgn5
Chaf1a
CHROMATIN
REMODELING
Chek1
cdc45
cdk1
Cenpp
CELL
CYCLE
Hist1h2bg
Hist1h2bn
Hist1h2b
Hist1h2bl
Hist1h2ak
Hist1h2bj
Hist1h2ba
HISTONES
Gata3
Irf4
TFs
WT
T_reg
Il2ra^mut/mut
T_regs
g
Foxp3
WT T_regs
Il2ra^mut/mut
T_regs
Ppp1r3f
Ppp1r3fos
Foxp3
Ccdc22

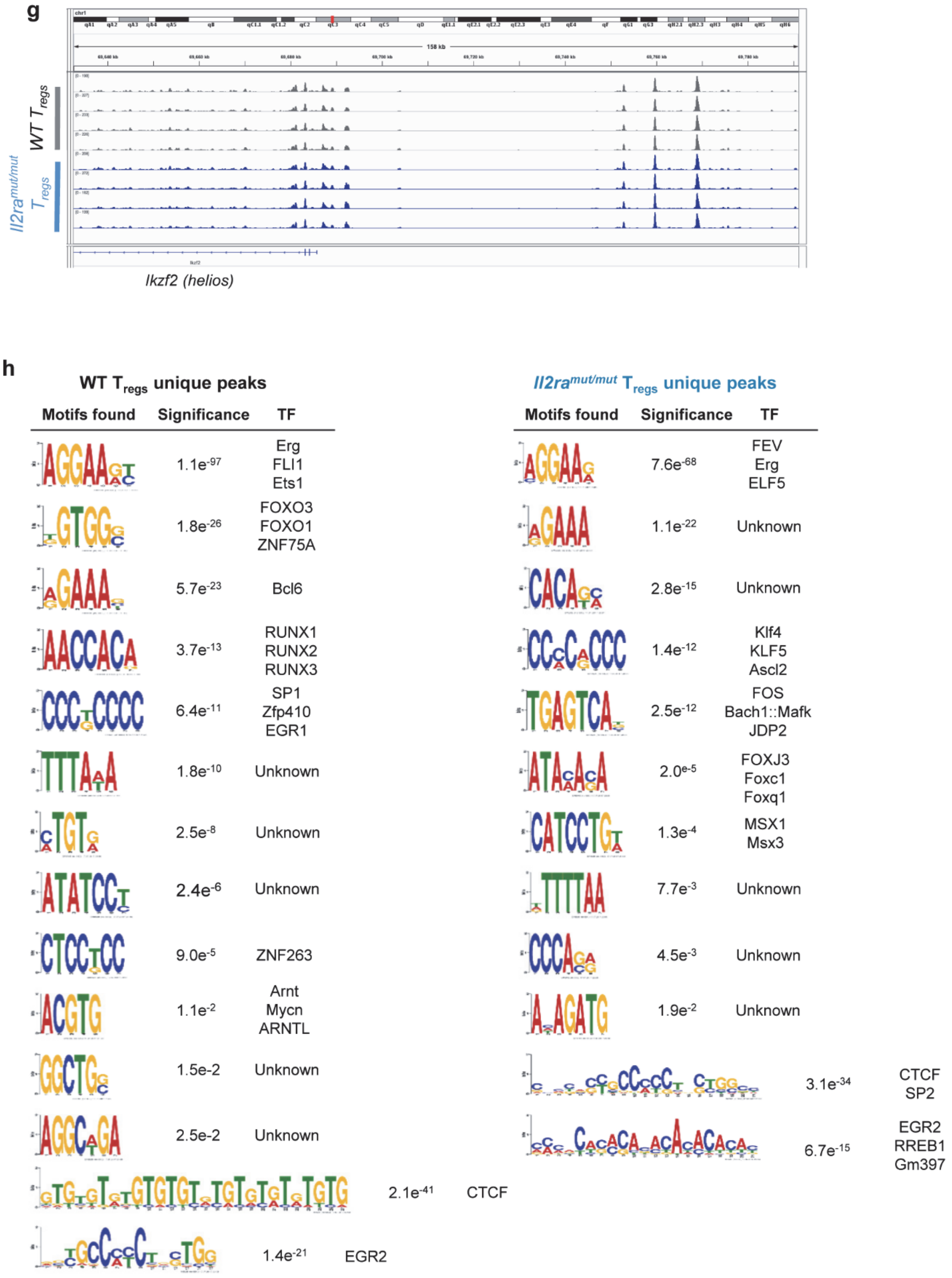

Supplementary Figure 4| Gene expression program of *Il2ramut/mut* versus WT Treg cells: 5x10[4] T_{reg} and T_{conv} cells were sorted by flow cytometry from LNs of 3 independent replicate *Il2ra[mut/mut]* or WT *Foxp3[Rfp/Rfp]* reporter mice and total RNA extracted and reverse transcribed to

cDNA. Affymetrix mouse expression arrays (Pico 1.0) were then conducted. **(a)** Scatter plot of individual genes expressed in *Il2ra*$^{mut/mut}$ versus WT T_{conv} cells. **(b)** Volcano plot of P-values versus gene expression fold change in *Il2ra*$^{mut/mut}$ versus WT T_{reg} cells. For **(a,b)** significantly up- and down-regulated genes were defined as genes with at least 1.5 fold change, p-value$\leq$ 0.01, and colored blue or black, respectively. **(c)** LN cells from 8 weeks old *Il2ra*$^{mut/mut}$ and WT mice were stained for cell surface CD3, CD4, intracellular Foxp3 and indicated markers. Overlay of representative FACS histograms after gating on $CD3^+CD4^+Foxp3^+$ T cells are shown and bar graphs average relative expression levels (% or MFI, n=5). **(d)** Network analysis of biological-process gene-ontology (BP-GO) term enrichment among significantly upregulated genes in *Il2ra*$^{mut/mut}$ vs. control WT T_{reg} cells. Upregulated genes were analyzed for over-represented GO terms using BiNGO in Cytoscape, and the resulting network was calculated and visualized using ReviGO. Node color is proportional to the FDR-adjusted P-value of the enrichment. Node size is proportional to gene set size. **(e)** Network analysis of BP-GO term enrichment among significantly upregulated genes in *Il2ra*$^{mut/mut}$ versus control WT T_{reg} cells. Upregulated genes were analyzed for over-represented GO terms using DAVID, and the resulting network was calculated and visualized using EnrichmentMap in Cytoscape. Groups of similar GO terms were manually circled. Line thickness and color are proportional to the similarity coefficient between connected nodes. Node color is proportional to the FDR-adjusted P-value of the enrichment. Node size is proportional to gene set size. **(f)** Heatmap representing genes related to chromosome modeling and maintenance that are differentially expressed in $Foxp3^+$ T_{reg} cells from *Il2ra*$^{mut/mut}$ compared to WT mice (p-value<0.05, Fold change >1.3, 3 mice/group). Color intensity represents Z-score values, i.e., variation to the mean. Dendrogram on the top dissociates samples between WT T_{reg} and *Il2r*$^{mut/mut}$ based on level expression for listed genes. **(g)** Analysis of OCRs in genes encoding for Foxp3 and Helios in thymic T_{reg} cells from WT versus *Il2ra*$^{mut/mut}$ mice. Individual ATAC-seq profiles of T_{reg} cells purified from 4 independent mice in each group is shown. **(h)** Enrichment of TF potential binding sites inside OCRs of WT versus *Il2ra*$^{mut/mut}$ T_{reg} cells using MEME-ChIP.

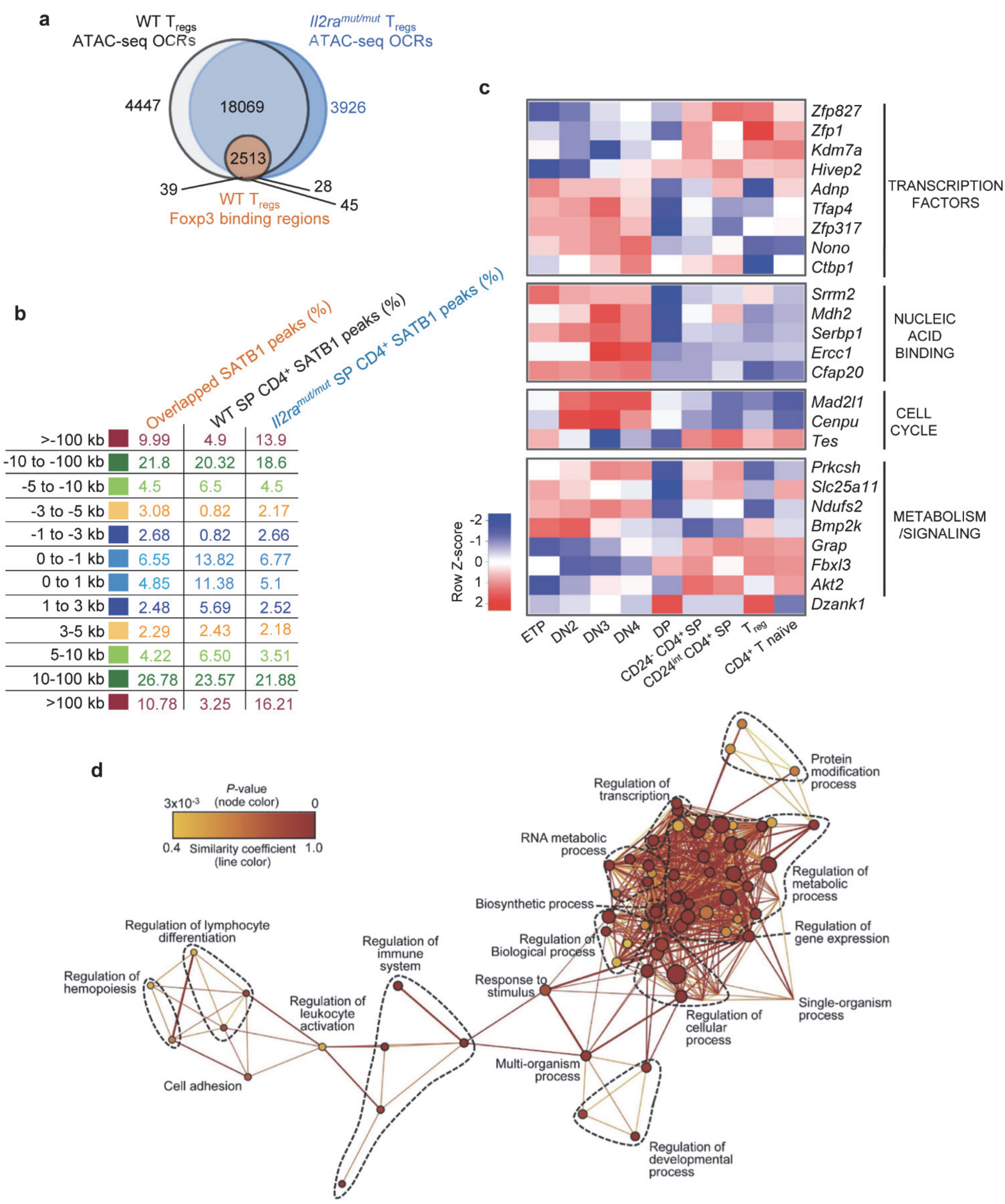

Supplementary Figure 5| Foxp3 binding does not account for IL-2-mediated epigenetic changes. (**a**) Three way Venn diagram comparing the number of common and unique ATAC-seq peaks in *Il2ra*$^{mut/mut}$ versus WT T_{reg} cells to FOXP3 DNA binding peaks in WT T_{reg} cells obtained from ChIP-seq of FOXP3 from Samstein et al. [4]. (**b**) Actual values for representation shown in

Figure 5c. **(c)** Relative expression of genes with unique SATB1 binding peaks in TSS (+/- 2kb) in WT SP $CD4^{+}$ T cells throughout thymocyte development using raw data from NCBI database. Row z-score is shown in heat map. **(d)** Network analysis of biological-process gene-ontology term enrichment among genes with unique SATB1 DNA binding peaks in $\textit{Il2ra}^{mut/mut}$ SP $CD4^{+}$ thymocytes. Genes with unique SATB1 binding peaks were analyzed for over-represented GO terms using BiNGO in Cytoscape, and the resulting network was calculated and visualized using EnrichmentMap in Cytoscape. Groups of similar GO terms were manually circled. Line thickness and color are proportional to the similarity coefficient between connected nodes. Node color is proportional to the FDR-adjusted P-value of the enrichment. Node size is proportional to gene set size.

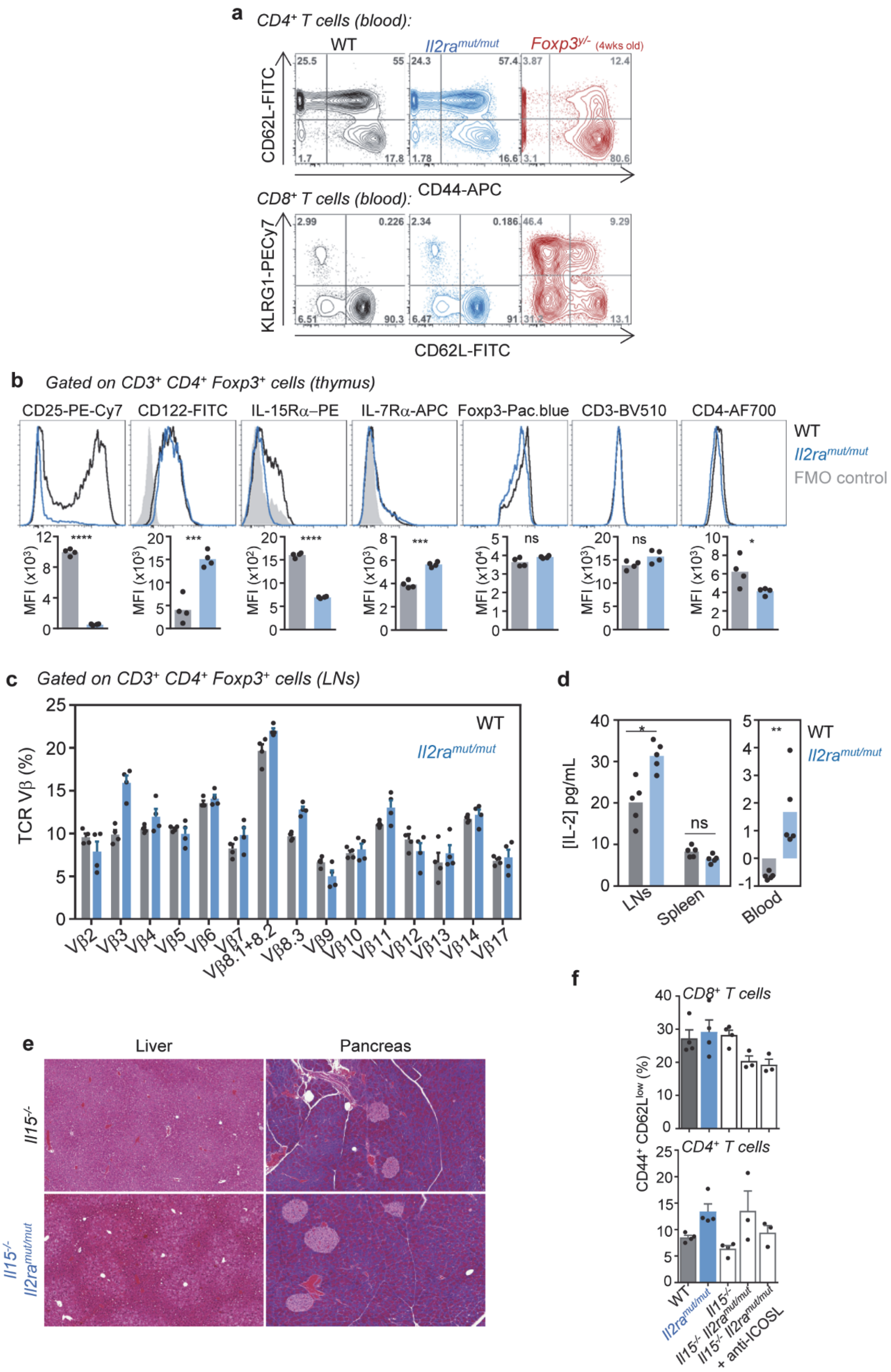

a
CD4+ T cells (blood):
WT
Il2ra mut/mut
Foxp3 y/- (4wks old)
CD62L-FITC
CD44-APC
CD8+ T cells (blood):
KLRG1-PECy7
CD62L-FITC
b
Gated on CD3+ CD4+ Foxp3+ cells (thymus)
CD25-PE-Cy7
CD122-FITC
IL-15Rα–PE
IL-7Rα-APC
Foxp3-Pac.blue
CD3-BV510
CD4-AF700
WT
Il2ra mut/mut
FMO control
MFI (x10³)
MFI (x10²)
MFI (x10⁴)
ns
c
Gated on CD3+ CD4+ Foxp3+ cells (LNs)
TCR Vβ (%)
WT
Il2ra mut/mut
Vβ2 Vβ3 Vβ4 Vβ5 Vβ6 Vβ7 Vβ8.1+8.2 Vβ8.3 Vβ9 Vβ10 Vβ11 Vβ12 Vβ13 Vβ14 Vβ17
d
[IL-2] pg/mL
ns
LNs
Spleen
Blood
WT
Il2ra mut/mut
e
Liver
Pancreas
Il15-/-
Il15-/- Il2ra mut/mut
f
CD8+ T cells
CD4+ T cells
CD44+ CD62L low (%)
WT
Il2ra mut/mut
Il15-/-
Il15-/- Il2ra mut/mut
Il15-/- Il2ra mut/mut + anti-ICOSL

Supplementary Figure 6| Characterization of *Il2ra*$^{mut/mut}$ mice. (**a**) Cell surface expression of indicated markers on $CD4^+$ and $CD8^+$ T_{conv} cells in the blood of *Il2ra*$^{mut/mut}$, WT (18 months) and *Foxp3*$^{y/-}$ (5 weeks) mice after birth. Dot plots are representative of 1 of 5 mice. (**b**) Thymocytes from 8-10 weeks old *Il2ra*$^{mut/mut}$ and WT mice were stained for cell surface CD3, CD4, intracellular Foxp3 and indicated markers. Overlay of representative FACS histograms after gating on $CD3^+CD4^+Foxp3^+$ T cells are shown and bar graphs average relative expression levels (MFI, n=4-5). (**c**) Relative proportion of LN $Foxp3^+$ T_{reg} cells expressing indicated TCR Vβ chains in *Il2ra*$^{mut/mut}$ and WT mice. (**d**) IL-2 levels measured by ELISA (eBioscience) in LNs, blood and spleens of *Il2ra*$^{mut/mut}$ and WT mice. (**e**) Liver and pancreas of *Il15*$^{-/-}$ and *Il15*$^{-/-}$*Il2ra*$^{mut/mut}$ mice (~6 months old) were harvested, fixed/cut and stained with H/E (n=6-7). (**f**) Proportion of activated ($CD44^+CD62L^{lo}$) peripheral blood T_{conv} cells among $CD3^+CD4^+$ or $CD8^+$ T cells in indicated mice treated (or not) with anti-ICOSL for 2 weeks. P-values are indicated when relevant with *$p < 0.05$; **$p< 0.01$; ***$p< 0.001$; NS, not significant, using two-tailed unpaired Student's t test.

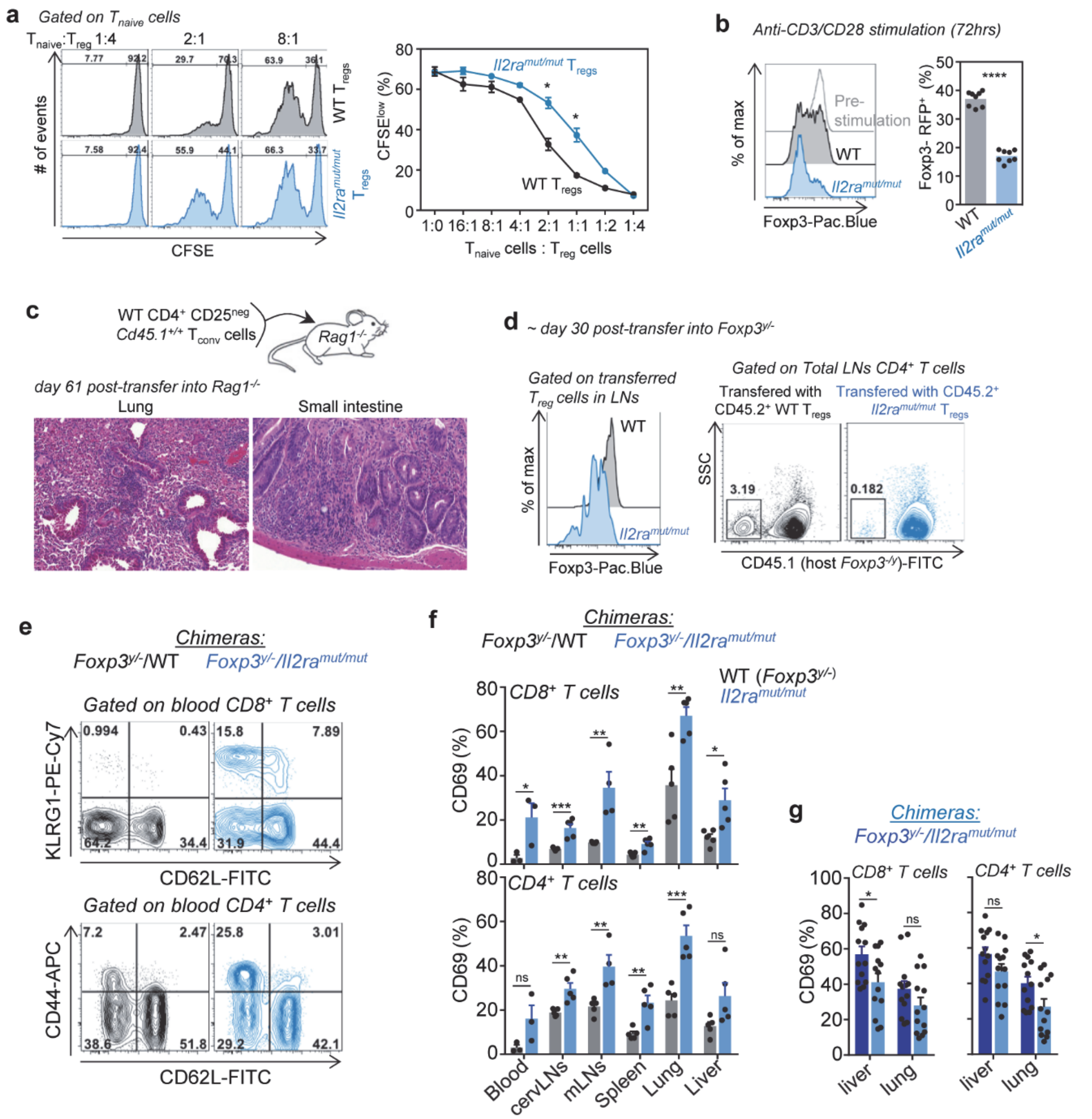

Supplementary Figure 7| Function and stability of *Il2ra*$^{mut/mut}$ T$_{reg}$ cells. (**a**) *In vitro* suppression assay comparing *Il2ra*$^{mut/mut}$ and WT T$_{reg}$ cells. CFSE-labeled CD4^{+} T$_{conv}$ cells were stimulated with anti-CD3 for 72 h in presence of irradiated splenocytes and indicated ratios of sorted *Il2ra*$^{mut/mut}$ or WT T$_{reg}$ cells. Representative FACS histogram of CFSE dilutions are shown with a summary plot of proliferated T$_{conv}$ cells (% CFSE^{lo}) of 4 independent replicate experiments. (**b**) Foxp3 expression (RFP) in anti-CD3 stimulated T$_{reg}$ cells (72 h) in a representative FACS histogram with summary bar graph (each symbol is 1 individual mouse). (**c**) Pictures of H/E stained sections of lung and small intestine from *Rag1*$^{-/-}$ mice transferred with WT CD4^{+}CD25^{neg} T$_{conv}$ cells only. (**d**) Fate mapping and Foxp3 expression of *Il2ra*$^{mut/mut}$ or WT Foxp3^{+} T$_{reg}$ cells into

Foxp3$^{y/-}$ mice 30 days post-transfer. (**e**) Cell surface expression of indicated activation markers of blood $CD4^+$ and $CD8^+$ T_{conv} cells from mixed *Foxp3*$^{y/-}$/ *Il2ra*$^{mut/mut}$ and *Foxp*$^{y/-}$/ WT bone marrow chimera mice ~2 months post reconstitution. Representative FACS dot plots of 1 of 5-9 mice are shown. (**f, g**) Proportion of CD69 expressing $CD4^+$ and $CD8^+$ T_{conv} cells in *Foxp3*$^{y/-}$/ *Il2ra*$^{mut/mut}$ and *Foxp3*$^{y/-}$/ WT chimeras (f) and of *Il2ra*$^{mut/mut}$ $CD4^+$ and $CD8^+$ T_{conv} cells in mixed *Foxp3*$^{y/-}$/ *Il2ra*$^{mut/mut}$ (g) chimeras throughout indicated organs ~2 months post reconstitution. P-values are indicated when relevant with *$p < 0.05$; **$p < 0.01$; ***$p < 0.001$; NS, not significant, using two-tailed unpaired Student's t test.

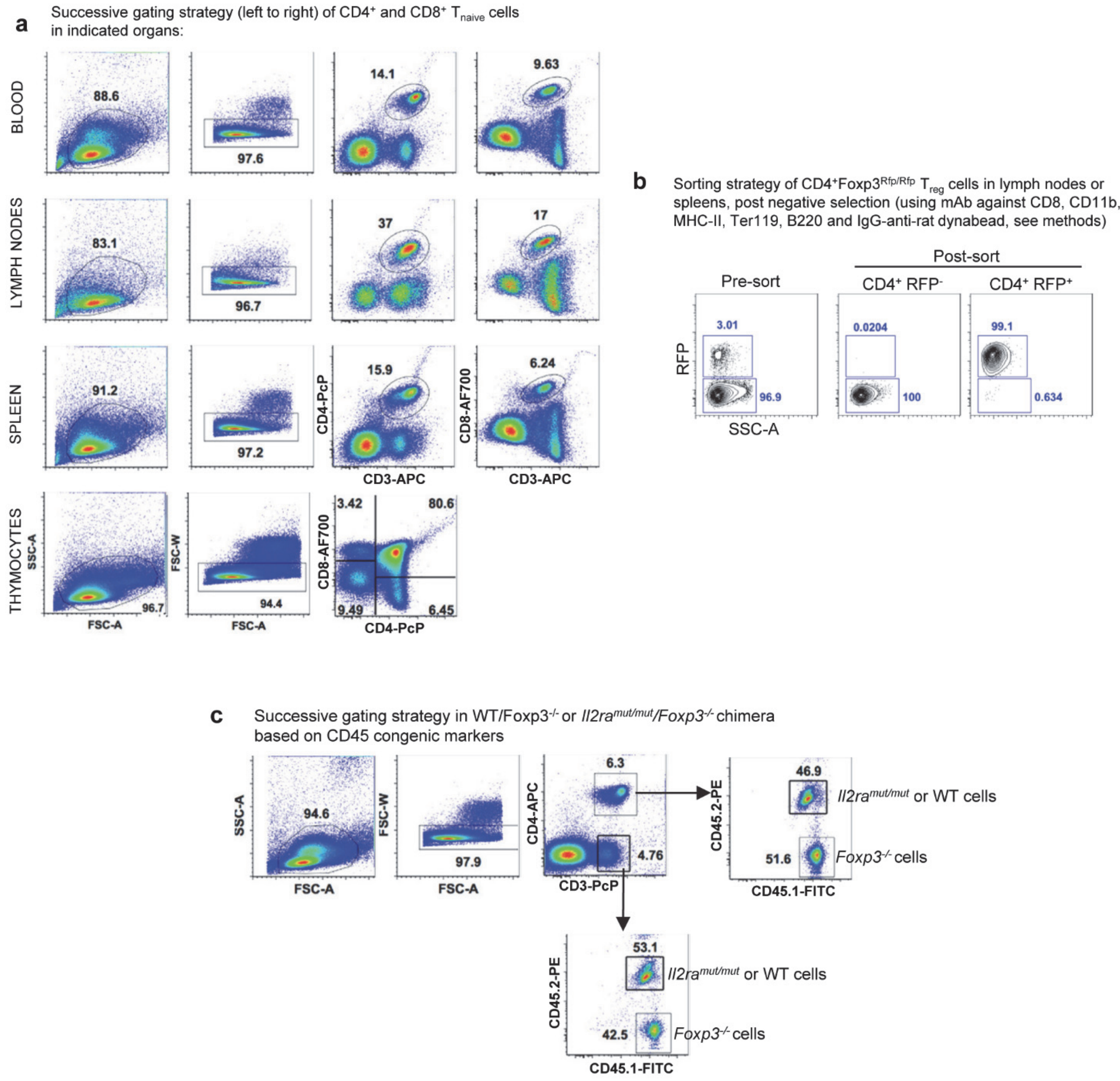

Supplementary Figure 8|. FACS sequential gating and sorting strategies used in the study. (**a**) Gating strategy for FACS data shown in Figs 1a, 1b, 1h, 2a-e, 6a, 6c, 6d, 6f, 7d, 7e and Supplementary Figs 1a-d, 2b-d, 6a-d, 6f, 7d. (**b**) Sorting gates (Aria III) of WT or *Il2ra*$^{mut/mut}$ *Foxp3*$^{Rfp/Rfp}$ T_{reg} cells for data shown in Figs 3-5 and Supplementary Figs 3-5 and 7a, 7b. (**c**) Gating strategy for FACS data of mixed bone marrow chimeras shown in Figs 7f-h, 7j, 7k and Supplementary Figs 7e-g.

Supplementary references

1. van der Veeken, J. *et al.* Memory of Inflammation in Regulatory T Cells. *Cell* **166**, 977-990 (2016).

2. Scott-Browne, J.P. *et al.* Dynamic Changes in Chromatin Accessibility Occur in CD8+ T Cells Responding to Viral Infection. *Immunity* **45**, 1327-1340 (2016).

3. Sen, D.R. *et al.* The epigenetic landscape of T cell exhaustion. *Science* **354**, 1165-1169 (2016).

4. Samstein, R.M. *et al.* Foxp3 exploits a pre-existent enhancer landscape for regulatory T cell lineage specification. *Cell* **151**, 153-166 (2012).

Reagent/Antibody	Fluorochrome	Clone	Purchased from	Dilution used
**FACS**				
anti-CD8α	FITC, PE, PcP, APC, Alexa 700, BV510, PE-Cy7	53-6.7	eBiosciences	1/400
anti-CD4	FITC, PE, APC, Alexa 700	RM4-5	eBiosciences	1/400
anti-CD3	Alexa 488 , BV510, PcP	145-2C11 or 17A2	eBiosciences	1/150
anti-CD45	Alexa 700	30F11	eBiosciences	1/200
anti-CD45.1	FITC, PE, Pacific Blue	A20	eBiosciences	1/200
anti-CD25	PE-Cy7	PC61	eBiosciences	1/300
anti-CD25	Biotin	7D4	BD Biosciences	1/500
anti-CD45.2	Alexa 700, PE, APC	104	eBiosciences	1/200
anti-CD40	APC, PE	1C10	eBiosciences	1/200
anti-CD80	FITC	1615A1	eBiosciences	1/200
anti-CD44	APC, Alexa 700	IM7	eBiosciences	1/400
anti-CD62L	FITC, PcP,	MEL-14	BD Biosciences	1/500
anti-CTLA4	APC	MIG2F5.5	eBiosciences	1/400
anti-GITR	biotin	DTA-1	BD Biosciences	1/800
anti-KLRG1	PE-Cy7	2F1	BD Biosciences	1/300
anti-ICOS	APC	C3984A	eBiosciences	1/400
anti-CD122 (IL-2Rβ)	FITC	TM-b1	BD Biosciences	1/200
anti-IL-15rα	PE	DNT15rα	BD Biosciences	1/200
anti-CD127 (IL-7Rα)	APC	A7R34	BD Biosciences	1/150
anti-Foxp3	Pacific Blue	FJK165	BD Biosciences	1/300
anti-Ki67	FITC, APC	SolA15	eBiosciences	1/200
anti-P-STAT5 (pY694)	PE	47/Stat5 (pY694)	BD Biosciences	5μL/sample
anti-TCT Vβ screening panel	FITC	N.A.	BD Biosciences	1/200
anti-CCR5	biotin	C34-3448	BD Biosciences	1/200
anti-CCR4 (CD194)	PE	2G12	BioLegend	1/200
anti-TIGIT	PE	1G9	BD Biosciences	1/200
anti-CD272 (BTLA)	PE	HMBT-6B2	BD Biosciences	1/200
anti-IL-2	eFluor 450	JES6-5H4	eBiosciences	1/200
anti-IFNγ	PE, PcP	XMG1.2	eBiosciences	1/300
anti-IL-17A	PE	TC11-18H10	BD Biosciences	1/200
streptavidin	PE, APC, Pacific Blue, Alexa 700, FITC		eBiosciences	1/500 - 1/1000
anti-goat	Alexa 488 , BV510, PcP	Polyclonal (Donkey)	Invitrogen	1/500
anti-rabbit	Alexa 546	Polyclonal (Donkey)	Invitrogen	1/500
**Fluorescent microscopy**				
anti-calreticulin		Polyclonal (Rabbit)	Thermo Fisher	1/300
anti-CD25		Polyclonal (Goat)	R and D System	1/300
**In vitro stimulation**				
anti-CD3ε	Purified	145-2C11	BD Biosciences	10μg/mL
**In vitro depletion (negative selection)**				
anti-CD4		GK1.5	eBioscience	5μg/mL
anti-B220		RA3-6B2	eBioscience	5μg/mL
anti-MHCII		M5/114	eBioscience	5μg/mL
anti-CD11b		M1/70	eBioscience	5μg/mL
anti-CD8β		H35	eBioscience	5μg/mL
anti-Ter119		Ter119	eBioscience	5μg/mL
**In vivo depletion**				
anti-CD8β		H35	home made	150μg/mouse
anti-CD4		GK1.5	home made	150μg/mouse

Supplementary Table 1| List of mAbs used in the study.
