# Supplementary material for: Interleukin 2 modulates thymic-derived regulatory T cell epigenetic landscape
Source: Nat Commun. 2018 Dec 18;9:5368. doi: 10.1038/s41467-018-07806-6 (PMC6299086; doi:10.1038/s41467-018-07806-6)
Supplement: Supplementary file 3 — Description of Additional Supplementary Files [file 41467_2018_7806_MOESM3_ESM.pdf]

**Description of Additional Supplementary Files :**

**Supplementary Data File 1 (related to Fig. 3b)**| List of common and unique ATAC-seq peaks in WT and *Il2ra*$^{mut/mut}$ thymic and LN $T_{reg}$ cells across gene organization.

**Supplementary Data File 2 (related to Fig. 3d)**| List of genes with unique peaks in WT versus *Il2ra*$^{mut/mut}$ thymic and LN $T_{reg}$ cells.

**Supplementary Data File 3 (related to Fig. 3e and Sup Fig. 3d)**| List of Biological Process Gene Ontology (BP-GO) from genes with unique peaks (OCRs) in WT versus *Il2ra*$^{mut/mut}$ thymic and LN $T_{reg}$ cells (all and 20kb from TSS).

**Supplementary Data File 4 (related to Fig. 4c)**| List of genes up and downregulated in WT versus *Il2ra*$^{mut/mut}$ $T_{reg}$ cells.

**Supplementary Data File 5 (related to Fig. 4d and Sup Fig. 4d,e)**| List of Biological Process Gene Ontology (BP-GO) from genes upregulated in *Il2ra*$^{mut/mut}$ versus WT $T_{reg}$ cells.

**Supplementary Data File 6 (related to Fig. 4e,g)**| List of differentially expressed genes in WT versus *Il2ra*$^{mut/mut}$ LN $T_{reg}$ cells compared to genes with unique peaks in WT and *Il2ra*$^{mut/mut}$ $T_{reg}$ cells within 500 bp from TSS.

**Supplementary Data File 7 (related to Fig. 5d)**| List of unique genes with (i) SATB1 peaks in WT SP $CD4^+$ thymocytes, (ii) SATB1 peaks in *Il2ra*$^{mut/mut}$ SP $CD4^+$ thymocytes and (iii) common genes with unique SATB1 peaks in WT and *Il2ra*$^{mut/mut}$ SP $CD4^+$ thymocytes.

**Supplementary Data File 8 (related to Fig. 5e,f)**| BP-GO terms of unique genes with SATB1 peaks in WT and in *Il2ra*$^{mut/mut}$ SP $CD4^+$ thymocytes.
