# Supplementary material for: Interleukin 2 modulates thymic-derived regulatory T cell epigenetic landscape
Source: Nat Commun. 2018 Dec 18;9:5368. doi: 10.1038/s41467-018-07806-6 (PMC6299086; doi:10.1038/s41467-018-07806-6)
Supplement: Supplementary file 2 — Peer Review File [file 41467_2018_7806_MOESM2_ESM.pdf]

Reviewers' comments:

Reviewer #1 (T reg, epigenetic)(Remarks to the Author):

1. The authors looked at the difference of STAT5 phosphorylation in Treg cells from thymus and LNs and in CD4+ Tconv from Il2ramut/mut mice. Could they specify whether it is STAT5A or STAT5B that was examined?
2. Impaired IL-2 signaling was achieved thru the Il2ramut/mut mice model. What would be the impact of elevated IL-2 signaling on Treg cells, e.g. thru administration of exogenous IL-2?
3. Regarding the Suppression assay: under strong TCR signal, wildtype Treg cells may not function normally either. Were different TCR signal strengths tested? If so, What was the threshold?
4. It would be important to discuss the lack of impact of these findings on induced-Treg cells.
5. Examining this in human thymic derived T cells with CD25 deficiency or with CD25 blockade could be performed.
6. No mention of limitations occurred in the discussion. There are several limitations since they have not ruled out other pathways, have not found a critical role for SATB1 in all Treg (so their title should be changed), and have not examined the applicability to humans and human disease.
7. A recent article (Nat Immunol. 2017 Oct;18(10):1160-1172.) reported Treg cells have individual, tissue-specific features in DNA-methylation landscapes (e.g., in LN, thymus, fat, and skin). Foxp3 methylation pattern in induced Treg cells differs from that of naïve Treg cells. How do you incorporate your results with their previous findings?
8. There were a number of places in the MS where grammar needed to be revised. For ex/ ‘wondered’ (p 11) is not scientifically precise language. Another ex/ “signals acts rather than signals act:. Another ex/ Treg cells ‘indexed’ to a low IL-2 Receptor? What does this mean?
9. There is no mention of the CNS1 modifications that can affect Treg.
10. It would be helpful for them to probe their autoimmune models with greater depth to understand mechanisms of IL-2 signaling over time during development of the immune system.

Reviewer #2 (Treg, TCR signaling, tolerance)(Remarks to the Author):

Chorro et al. identify and characterize a naturally-occurring loss-of-function IL2ra mutant mouse (IL2ramut/mut). While the contribution of CD25 to Treg cell function in the periphery has been published, the loss of function mutation results in a novel phenotype as no spontaneous inflammatory disease is observed despite a decrease in Treg function to control WT Teff cells in transfer models. Moreover, they show that IL-2 signals are involved in shaping the chromatin accessibility of Tregs. Overall, this work would be of interest to the scientific community. The following concerns can be addressed with revisions to soften/clarify the text.

1. One concern Is that they do not follow up the epigenetic data. While SATB1 could be inferred to perform the functions downstream of IL-2 based on correlation analysis, this is not experimentally tested, making the “via the pioneer factor SATB1” in the title a bit strong in my opinion. The mechanism by which epigenetic changes occur in IL2ramut/mut Treg cells involving ectopic binding of the transcription factor SATB1 is not explored. I think it would be suitable to edit the text to soften the language.

2. The authors set out to study the role of IL-2 signaling in thymic Treg cell development, yet their analyses of OCRs and gene expression in peripheral Foxp3+ Treg cells may reflect effects post development.

The authors use Fig. 5 to suggest that the effects of IL-2 occur prior to Treg selection as they analyzed

CD4SP. First, is the entire CD4SP population used? The labeling in Fig. 5 indicates Tregs and not CD4SP. However, If other subsets normally CD25 expressing (Treg/CD25+ Foxp3-) are included in the CD4SP, the data may reflect changes in those subsets rather than a global effect of CD25mut on cells that are in a state prior to Treg selection.

3. In the mixed bone marrow chimeras (Fig. 2e), what was the ratio of mut vs WT Tregs in the thymus?

4. Numerous errors/need for clarification.
Figure referenced after “likely ruling out a microbiota hypothesis” should be Supplementary Figure 1d, not Fig. 1d (page 5).

Fig7b. Rag2 (legend) or Rag1 (Figure) KO?

Figure referenced after “CTV dilution of labelled T cells in response to anti-CD3 and varied amounts of IL-2 should be Fig 2c, not Fig 3c (page 7).

Figure 4f ATAC-seq tracks are not labeled for WT and IL2ramut/mut mice. Assuming the bottom blue tracks are IL2ramut/mut mice, the highlighted peaks do not look larger for Gzmb and Prdm1.

The text states that “a different set of TFs such as FOXO, RUNX and KLF family members, could possibly bind in WT but not IL2ramut/mut Treg cells (Supplementary Fig. 4g), yet supplementary Figure 4g shows that a Klf binding motif is enriched in IL2ramut/mut Treg cells, not WT cells.

Figure referenced after “29 of the 96 genes bound in WT thymocytes were within less than 2 kb from the TSS” is listed as Fig. 5d, which seems incorrect (page 11).

The representation of enriched biological-process GO terms in Figure 3e is not particularly intuitive to compare mut vs WT.

The **major limitation point** in the main conclusion of our paper that was raised by both reviewers (**points#1, 2, 6, 10**) relates to the stage at which IL-2 alters thymic-derived T_{reg} cell epigenetic imprinting. The major message the study conveys is that IL-2 acts as an important modulator of $Foxp3^+$ T_{reg} cell-epigenetic landscape ; yet the initial version of our paper provided limited information as to when this IL-2-modulated epigenetic signature of T_{reg} cells may be acquired. We reported that in single positive (SP) $CD4^+$ thymocytes isolated from WT versus *Il2ra*$^{mut/mut}$ mice, the pioneer factor SATB1 is differentially positioned suggesting that such differences may already be set at the earliest stages of (SP) $CD4^+$ thymocyte commitment to the T_{reg} cell lineage. To formally test this possibility and strengthen the essential message of our work, we assessed whether or not T_{reg} cell-committed thymocytes from WT versus *Il2ra*$^{mut/mut}$ mice exhibited epigenetically distinct landscapes. We sorted thymic $Foxp3^+$ T_{reg} cells from either mice, and conducted ATAC-seq analysis (**Fig. 3**). We now provide a new set of results (**new Fig. 3**) revealing that the OCRs in $Foxp3^+$ thymic T_{reg} cells from WT compared to *Il2ra*$^{mut/mut}$ mice are already significantly different. Thus, these new data further support the proposed model that IL-2 dependent modulation of $Foxp3^+$ T_{reg} cell epigenetic signature already occurs during thymic development at the time thymocytes commit to the T_{reg} cell lineage.

II. Response to specific Reviewers' comments:

Reviewer #1 (T reg, epigenetic)(Remarks to the Author):

1. The authors looked at the difference of STAT5 phosphorylation in Treg cells from thymus and LNs and in CD4+ Tconv from Il2ramut/mut mice. Could they specify whether it is STAT5A or STAT5B that was examined?

We have used the clone 47/Stat5 (pY694) as indicated in the Supplementary information Ab table. This clone is specific for STAT5a, and we have now also added this information to the revised text.

2. Impaired IL-2 signaling was achieved thru the Il2ramut/mut mice model. What would be the impact of elevated IL-2 signaling on Treg cells, e.g. thru administration of exogenous IL-2?

The *Il2ra*$^{mut/mut}$ mice have elevated circulating/LN levels of IL-2 (Fig. S6d), most likely as the result of impaired consumption by the mutant IL-2 receptor, thus such exogenous IL-2 treatment alone is unlikely to have any impact. IL-2 signals may be restored by treatment with anti-IL-2/IL-2 complexes to bypass the need for IL-2Rα-dependent signals. Unfortunately, this treatment also induces strong T cell proliferation and an autoimmune-like syndrome after only a few injections (our data not shown), complicating interpretation of these experiments. Another potential model is the mouse expressing the constitutively active (CA) form of STAT5 developed by the Farrar lab (Burchill et al., J Immunol 2003). This has been extensively studied and shown

to rescue Foxp3$^+$ T_{reg} cell development in constitutive/inducible *Il2ra$^{-/-}$ or Il2rb$^{-/-}$* mice and to prevent subsequent fatal autoimmunity (Burchill et al., J Immunol 2007; Yu et al., Immunity 2009 ; Fontenot et al., Nat Immunol 2005 ; Chinen et al, Nat Immunol 2016), suggesting that epigenetic programing of STAT5-CA expressing T_{reg} cells is likely to be comparable to that of WT counterparts. While we agree with this reviewer that the question of how much IL-2 signals is needed to rescue WT Foxp3$^+$ T_{reg} cell epigenetic landscape in *Il2ra$^{mut/mut}$* T_{reg} cells, is a very interesting one, rigorous analysis would require a whole new study in which with *Il2ra$^{mut/mut}$* T_{reg} cells are temporally induced to express STAT5-CA and concomitantly fate-mapped for epigenetic analysis. The new set of data discussed in our general response above provide additional evidence to establish more precisely at which stage of development IL-2 signals alters T_{reg} cell epigenetic signature.

3. Regarding the Suppression assay: under strong TCR signal, wildtype Treg cells may not function normally either. Were different TCR signal strengths tested? If so, What was the threshold?

We tested 1 μg/mL of anti-CD3ε, as stated in the Materials and Methods section. It is possible that decreasing TCR signals (i.e., the amount of anti-CD3ε) will further improve T_{reg} cell's ability to mediate suppression. The data presented in Fig. S7a repeated 4 times already show a modest but significant difference in the ability of WT versus *Il2ra$^{mut/mut}$* T_{reg} cells to suppress T_{conv} cell proliferation *in vitro* and at various ratios. The most important and relevant sets of data to validate our point, however, and illustrate the functional loss of *Il2ra$^{mut/mut}$* T_{reg} cells, is related to the various *in vivo* models of autoimmunity presented in Figs. 7a, 7b and 7g, in which we establish that *Il2ra$^{mut/mut}$* T_{reg} cells are unable to suppress WT T_{conv}-cell mediated wasting disease.

4. It would be important to discuss the lack of impact of these findings on induced-Treg cells.

We have not analyzed induced T_{reg} cells, therefore we cannot formally make this claim. However, given that our new data suggests IL-2 signals impact natural T_{reg} cells already at the time they are just committed to the T_{reg} cell lineage in the thymus, it is likely that induced Treg cells will not be impacted by this mechanism. We have also outlined this idea in the revised discussion.

5. Examining this in human thymic derived T cells with CD25 deficiency or with CD25 blockade could be performed.

While these experiments may theoretically be interesting, there are multiple issues related to them. First, it is a challenging and lengthy process to request access to such human material that would go much beyond the current three months revision timeline of this paper. Second, we are only aware of four patients that have been reported so far with loss of CD25 function as a result of truncation mutations in the *Il2RA* gene (Cardirola et al., Front. Immunol, 2018), and these patients suffer severe combined immunodeficiency with viral and bacterial infections, lymphoproliferation and several multi-organ autoimmune disorders, all consistent with the mouse model. As far as the *Il2ra* point mutation we describe which has enabled our study, to our knowledge it has never been reported neither in mice or humans. It may exist in humans, but such people would be unlikely to exhibit any specific disease phenotype based on our analysis in the mouse, and it seems to be the focus of another study. Using CD25 blockade as also suggested, is unlikely to be feasible *in vivo* in humans. Therefore, we do not think that we can address this point.

6. No mention of limitations occurred in the discussion. There are several limitations since they have not ruled out other pathways, have not found a critical role for SATB1 in all Treg (so their

title should be changed), and have not examined the applicability to humans and human disease.

We agree that our findings are focused on natural thymic-derived T_{reg} cells in mice, and that IL-2 may modulate T_{reg} cell epigenetic landscape via non-SATB1-dependent mechanisms. We have therefore revised the title of our paper and now discussed these limitations accordingly.

7. A recent article (Nat Immunol. 2017 Oct;18(10):1160-1172.) reported Treg cells have individual, tissue-specific features in DNA-methylation landscapes (e.g., in LN, thymus, fat, and skin). Foxp3 methylation pattern in induced Treg cells differs from that of naïve Treg cells. How do you incorporate your results with their previous findings?

We neither analyzed peripheral tissue- nor induced T_{reg} cells, and we did not perform any DNA methylation analysis on our samples. Hence, the direct comparison between our results and those from the paper cited by this reviewer cannot be done. The differentially methylated regions discussed in this report were mostly within the gene body regions, where DNA methylation status reflects the expression of the gene, but is unlikely to contribute to regulating its expression (Ball et al., Nat Biotech 2009, 27:361 ; Suzuki et al., Genome Research 2011, 21:1833). Yet, we still analyzed genes encoding for the Foxp3 and HELIOS TFs that are respectively expressed in all or only thymic T_{reg} cells and were reported by these authors to exhibit differential methylation patterns in tissue/induced T_{reg} cells. We checked whether OCRs between WT and *Il2ra*$^{mut/mut}$ thymic T_{reg} cells were different (See Figure below) but we did not detect any differences in OCRs in promoter or gene body for these genes, suggesting that the modulation of IL-2 signals in natural T_{reg} cells is unlikely to account for these specific modifications in methylation profiles.

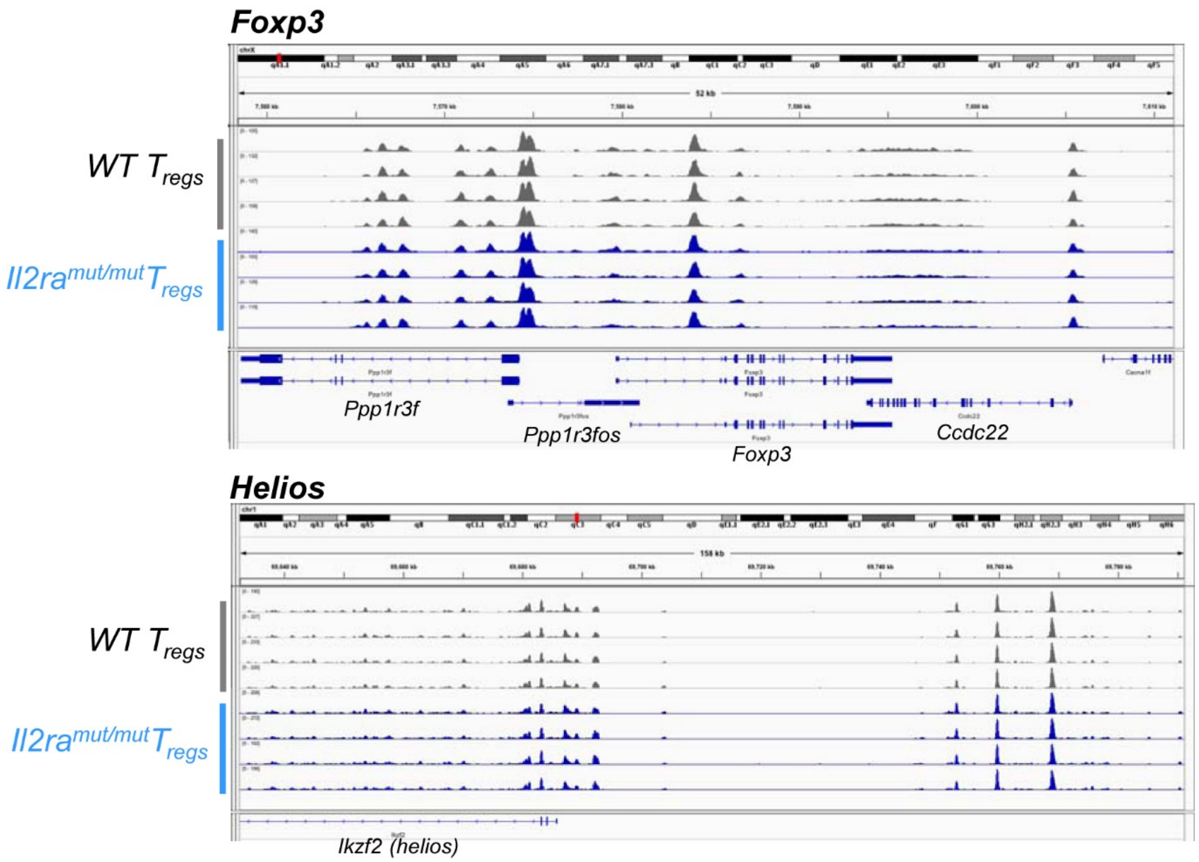

Analysis of OCRs in genes encoding for Foxp3 and HELIOS in thymic T_{reg} cells from WT versus *Il2ra*$^{mut/mut}$ mice. Individual ATAC-seq profiles of T_{reg} cells purified from 4 independent mice in each group is shown.

8. There were a number of places in the MS where grammar needed to be revised. For ex/ ‘wondered’ (p 11) is not scientifically precise language. Another ex/ “signals acts rather than signals act:. Another ex/ Treg cells ‘indexed’ to a low IL-2 Receptor? What does this mean?
We thanks this reviewer for pointing this out. We have addressed the specific comments above and carefully edited the revised manuscript. The “indexed” to a low IL-2 receptor concept came from the paper we cited and should have been quoted. We understand it as “being set to” which we have now used.

9. There is no mention of the CNS1 modifications that can affect Treg.
We did not find any OCR differences in the CNS1 of WT versus *Il2ra*$^{mut/mut}$ T_{reg} cells (See also our response in #7).

10. It would be helpful for them to probe their autoimmune models with greater depth to understand mechanisms of IL-2 signaling over time during development of the immune system.
The new Figure 3 presents further OCR analysis of natural T_{reg} cells at an earlier stage during their development. It strengthens the key message of this work, establishing a new role for IL-2 signals on T_{reg} cell epigenetic landscape at the earliest stage of thymocyte commitment to this lineage (See also “General comment” section above). In Figure 7, we present 3 different models of autoimmunity. The *Foxp3*$^{-/-}$ and the *Rag*$^{-/-}$ rescue/transfer models (7a & 7b) cannot be used to

study the development of the immune system. The radiation chimeras models (7f*: Foxp3*$^{-/-}$*/Il2ra*$^{mut/mut}$ and 7g: *Foxp3*$^{-/-}$*/Il2ra*$^{mut/mut}$) could theoretically be exploited, however, *Il2ra*$^{mut/mut}$ T_{reg} cells are lost in competition for IL-2 against WT T cells and Fig. 7g mice further develop rapid autoimmunity (4-6 weeks post reconstitution), also precluding their use to probe IL-2 signaling during the development of the immune system.

Reviewer #2 (Treg, TCR signaling, tolerance)(Remarks to the Author):

Chorro et al. identify and characterize a naturally-occurring loss-of-function IL2ra mutant mouse (IL2ramut/mut). While the contribution of CD25 to Treg cell function in the periphery has been published, the loss of function mutation results in a novel phenotype as no spontaneous inflammatory disease is observed despite a decrease in Treg function to control WT Teff cells in transfer models. Moreover, they show that IL-2 signals are involved in shaping the chromatin accessibility of Tregs. Overall, this work would be of interest to the scientific community. The following concerns can be addressed with revisions to soften/clarify the text.

1. One concern Is that they do not follow up the epigenetic data. While SATB1 could be inferred to perform the functions downstream of IL-2 based on correlation analysis, this is not experimentally tested, making the “via the pioneer factor SATB1” in the title a bit strong in my opinion. The mechanism by which epigenetic changes occur in IL2ramut/mut Treg cells involving ectopic binding of the transcription factor SATB1 is not explored. I think it would be suitable to edit the text to soften the language.
We acknowledge that we do not yet know the precise mechanism by which SATB1 induces epigenetic changes in natural T_{reg} cells. Thus we have edited and softened the title and the text of the revised manuscript.

2. The authors set out to study the role of IL-2 signaling in thymic Treg cell development, yet their analyses of OCRs and gene expression in peripheral Foxp3+ Treg cells may reflect effects post development.
This is an excellent point that we decided to address by conducting further analysis of the epigenetic landscape of mature Foxp3^{+} T_{reg} cells in the thymus from WT compared to *Il2ra*$^{mut/mut}$ T_{reg} cells (**new Figure 3**). The data show that already at this early stage of commitment of CD4^{+} SP thymocytes to the T_{reg} cell lineage, a significant difference in OCRs is noted, strengthening the key message of this work (See also “General comment” section above). Together with altered SATB1 positioning on SP CD4^{+} thymocytes, we believe that the impact of IL-2 on the epigenetic landscape of natural T_{reg} cells already occurs at early but not late stages of their development.

The authors use Fig. 5 to suggest that the effects of IL-2 occur prior to Treg selection as they analyzed CD4SP. First, is the entire CD4SP population used? The labeling in Fig. 5 indicates Tregs and not CD4SP. However, If other subsets normally CD25 expressing (Treg/CD25+ Foxp3-) are included in the CD4SP, the data may reflect changes in those subsets rather than a global effect of CD25mut on cells that are in a state prior to Treg selection.
We apologize for this labelling mistake. All “T_{regs}” label should read “SP CD4^{+} Thymocytes”, and this has now been corrected in the revised Figure 5. The thymocyte population that was analyzed in the SATB1 ChIP-seq experiments are SP CD4^{+} thymocytes including “pre-T_{reg} cells” that do not yet express Foxp3. The new epigenetic analysis of mature Foxp3^{+} thymic T_{reg} cells that we added in the new Figure 3 further shows that significant OCR differences were already present at a very early stage of thymocyte commitment to the T_{reg} cell lineage, as we reported in

the periphery. These data further support our proposed model that IL-2 impacts natural T_{reg} cell epigenetic landscape very early during their development.

3. In the mixed bone marrow chimeras (Fig. 2e), what was the ratio of mut vs WT Tregs in the thymus?

In Figure 2e, we never looked at the ratio of WT versus *Il2ra*$^{mut/mut}$ thymocytes since these mice were used for *Listeria*-Ova challenge experiments and WT versus *Il2ra*$^{mut/mut}$ Ova-specific T_{conv} cell expansion. The ratio of T_{conv} cells in the blood was 60 (WT):40 (*Il2ra*$^{mut/mut}$) both in all hematopoietic ($CD45^+$) and in the T cell compartments. This result was consistent across experiments in which mixed bone marrow chimeras were used (Fig. 7).

4. Numerous errors/need for clarification.

We thank this reviewer for pointing these out. We have corrected these mistakes and addressed all specific points below.

Figure referenced after "likely ruling out a microbiota hypothesis" should be Supplementary Figure 1d, not Fig. 1d (page 5).

This has been corrected.

Fig7b. Rag2 (legend) or Rag1 (Figure) KO?

This is *Rag1*$^{-/-}$ (JAX#002216), now corrected in the Figure 7 legend.

Figure referenced after "CTV dilution of labelled T cells in response to anti-CD3 and varied amounts of IL-2 should be Fig 2c, not Fig 3c (page 7).

This has been corrected.

Figure 4f ATAC-seq tracks are not labeled for WT and IL2ramut/mut mice. Assuming the bottom blue tracks are IL2ramut/mut mice, the highlighted peaks do not look larger for Gzmb and Prdm1.

We have provided better resolution of the differentially called peaks to help clarify interpretation of these data.

The text states that "a different set of TFs such as FOXO, RUNX and KLF family members, could possibly bind in WT but not IL2ramut/mut Treg cells (Supplementary Fig. 4g), yet supplementary Figure 4g shows that a Klf binding motif is enriched in IL2ramut/mut Treg cells, not WT cells.

This was a mistake which has now been corrected.

Figure referenced after "29 of the 96 genes bound in WT thymocytes were within less than 2 kb from the TSS" is listed as Fig. 5d, which seems incorrect (page 11).

This is the correct information. Among the 96 genes to which SATB1 uniquely binds in WT SP $CD4^+$ thymocytes, 29 are within less than 2 kb from the TSS. Of these 29 genes, expression of 24 of them that was available at different stages of thymocyte development (GEO GSE15907) are shown in Sup. Figure 5c. We have rewritten and clarified this section.

The representation of enriched biological-process GO terms in Figure 3e is not particularly intuitive to compare mut vs WT.

We agree and have now used a more synthetic representation of these data.

REVIEWERS' COMMENTS:

Reviewer #1 (Remarks to the Author):

The authors have addressed previous comments point by point, completed additional experiments achievable in the timeline given, and clarified several errors in the previous manuscript.

In this revision ATAC-seq analysis was used on sorted thymic Foxp3+ Treg cells from both groups to demonstrate the difference in OCRs. The results appear to be valid and the methodology is appropriate.

Overall, their work is nicely presented, and their findings bring new knowledge to the field.

No further comments for revision.

Reviewer #2 (Remarks to the Author):

The authors have adequately addressed my concerns.

Reviewer #1 (Remarks to the Author):
The authors have addressed previous comments point by point, completed additional experiments achievable in the timeline given, and clarified several errors in the previous manuscript.
In this revision ATAC-seq analysis was used on sorted thymic Foxp3+ Treg cells from both groups to demonstrate the difference in OCRs. The results appear to be valid and the methodology is appropriate.
Overall, their work is nicely presented, and their findings bring new knowledge to the field.
No further comments for revision.

Reviewer #2 (Remarks to the Author):
The authors have adequately addressed my concerns.
